# Crowdsourced direct-to-consumer genomic analysis of a family quartet

**DOI:** 10.1186/s12864-015-1973-7

**Published:** 2015-11-07

**Authors:** Manuel Corpas, Willy Valdivia-Granda, Nazareth Torres, Bastian Greshake, Alain Coletta, Alexej Knaus, Andrew P. Harrison, Mike Cariaso, Federico Moran, Fiona Nielsen, Daniel Swan, David Y. Weiss Solís, Peter Krawitz, Frank Schacherer, Peter Schols, Huangming Yang, Pascal Borry, Gustavo Glusman, Peter N. Robinson

**Affiliations:** The Genome Analysis Centre, Norwich Research Park, Norwich, UK; Orion Integrated Biosciences Inc., POX 028, Larchmont, NY 10538 USA; Universidad de Navarra, Grupo de Fisiología del Estrés en Plantas (Dpto. de Biología Ambiental), Pamplona, Spain; Department for Applied Bioinformatics, Institute for Cell Biology and Neuroscience, Goethe University, Frankfurt am Main, Germany; InSilico Genomics S.A., Avenue Adolphe Buyl, 87, Building C, 5th floor, B-1050 Brussels, Belgium; Institute for Medical Genetics and Human Genetics, Charité Universitätsmedizin Berlin, Berlin, Germany; Department of Mathematical Sciences, University of Essex, Wivenhoe Park, Colchester, Essex CO4 3SQ UK; SNPedia, River Road Bio, LLC, 9812 Falls Road #114-237, Potomac, Maryland, MD 20854 USA; Facultad de Ciencias Químicas, Universidad Complutense de Madrid, 28040 Madrid, Spain; DNADigest, Windsor House, Station Court, Station Road, Great Shelford, Cambridge, CB22 5NE UK; Oxford Gene Technology, Woodstock Road, Begbroke, Oxfordshire, OX5 1PF UK; GeneTalk, Finckensteinallee 84, 12205 Berlin, Germany; BIOBASE, Halchtersche Strasse 33, D-38304 Wolfenbuettel, Germany; Diploid Genomics, Middelweg 129, 3001 Leuven, Belgium; BGI, Main Building, Beishan Industrial Zone, Yantian District, Shenzhen, 518083 China; Department of Public Health and Primary Care, Catholic University of Leuven, Leuven, Belgium; The Institute for Systems Biology, 401 Terry Ave N, Seattle, WA 98109 USA; Berlin Brandenburg Center for Regenerative Therapies, Charité Universitätsmedizin Berlin, Berlin, Germany; Institut de Recherches Interdisciplinaires et de Developpements en Intelligence Artificielle, the Computer and Decision Engineering Department, Universite Libre de Bruxelles, 87 av. Adolphe Buyl, Bruxelles, 1050 Belguim

**Keywords:** Crowdsourcing, Personal genomes, Participatory medicine, SNPs, Exome analysis, Microbiome, Family genomes, Direct-to-consumer, Genetic testing, Genomics

## Abstract

**Background:**

We describe the pioneering experience of a Spanish family pursuing the goal of understanding their own personal genetic data to the fullest possible extent using Direct to Consumer (DTC) tests. With full informed consent from the Corpas family, all genotype, exome and metagenome data from members of this family, are publicly available under a public domain Creative Commons 0 (CC0) license waiver. All scientists or companies analysing these data (“the Corpasome”) were invited to return results to the family.

**Methods:**

We released 5 genotypes, 4 exomes, 1 metagenome from the Corpas family via a blog and figshare under a public domain license, inviting scientists to join the crowdsourcing efforts to analyse the genomes in return for coauthorship or acknowldgement in derived papers. Resulting analysis data were compiled via social media and direct email.

**Results:**

Here we present the results of our investigations, combining the crowdsourced contributions and our own efforts. Four companies offering annotations for genomic variants were applied to four family exomes: BIOBASE, Ingenuity, Diploid, and GeneTalk. Starting from a common VCF file and after selecting for significant results from company reports, we find no overlap among described annotations. We additionally report on a gut microbiome analysis of a member of the Corpas family.

**Conclusions:**

This study presents an analysis of a diverse set of tools and methods offered by four DTC companies. The striking discordance of the results mirrors previous findings with respect to DTC analysis of SNP chip data, and highlights the difficulties of using DTC data for preventive medical care. To our knowledge, the data and analysis results from our crowdsourced study represent the most comprehensive exome and analysis for a family quartet using solely DTC data generation to date.

**Electronic supplementary material:**

The online version of this article (doi:10.1186/s12864-015-1973-7) contains supplementary material, which is available to authorised users.

## Background

Direct-to-consumer (DTC) genetic testing has made it possible for the general public to access personal genomics information. A new sector has thus arisen in the biotechnological industry capitalising on selling genomic tests directly to the public, circumventing the need for consulting with a clinician before taking a test. This has encouraged the growth of P4 medicine (personalised, predictive, preventive and participatory) [1–3] whereby patients can take control of their own health and may be empowered to understand potential health problems even before symptoms arise. The Personal Genomes Project [4] also supports participation of healthy individuals in genomics research by providing a robust framework for sharing their data publicly with a unique consent process. The rapid development of the field, initially fuelled by companies such as 23andMe, deCODE Genetics, and Navigenics, has enabled sharing of data and development of methods for personal genomics analysis. However, this has come at the price of ever increasing demands on the breadth of knowledge and skills needed to interpret genetic risks and the consequent burden on DTC providers to keep up to date with scientific research. The value of DTC predictions has recently been questioned by the FDA, which has prohibited 23andMe from providing clinically related inferences based on their customers’ genotype. DTC providers cannot offer all available methods and scientific insights for any given set of genetic markers. This can be particularly frustrating for users who may want to explore further the raw data from which DTC predictions are made or simply apply third party tools to the analysis of their personal genomes themselves.

The power of family-based genomic analysis has been established [5–8]. DTC companies currently offer very limited support for genetic analyses involving more than one individual, e.g., whole families [5, 9]. Privacy considerations present additional challenges in family analyses, since different individuals of a family may have different comfort levels about sharing some or all of their personal genetic risks and data. In addition, this type of information might have consequences for offspring and children. Since members of a same family share much of their genetic sequence (and hence information), decisions made by one individual may affect the whole family. Hence, family-based personal genomics analyses may necessitate the re-evaluation of pre-established notions of identity and privacy, and of data ownership.

Here we present the personal genomics analysis of a set of exomes from a family (the Corpas family), and a microbiome analysis of an individual of this family. We build from our crowdsourcing on the previously published analysis of five samples of the Corpas family using 23andMe SNP chips [5]. We use DTC genetic testing with the goal of exploring the process of participatory medicine performed at the family level with current technology. One of the family members is a scientist – the bioinformatician leading this study (Manuel Corpas). After discussing privacy issues, the family made a group decision to make their personal genomics data publicly accessible and shareable. The family made their genome data available under a Creative Commons 0 (CC0) license waiver, with the underlying assumption that, by making their genomic data freely available, the chances of receiving analysis results from scientists and companies would be increased. Publicly sharing personal genomes has been reported as one of the driving factors for participants in open data projects for DTC genetic testing (e.g., openSNP [10]). They thus started a crowdsourcing project [11] to attract the attention of potential collaborators who might be able to analyse the data and return results that could complement their existing knowledge about their personal genomes. To emulate the principles of participatory medicine, the current study was designed to follow these principles: 1) all experiments must be carried out with private funds and as a private effort (i.e., using no public grant money, donations); 2) all individuals involved need to provide informed consent; 3) analyses are to be carried out by combining results from personal investigation and crowdsourcing efforts and 4) whenever possible, advice is to be taken from professional genetic counsellors and clinicians should any predicted risk require it. Once consent was provided, saliva samples were extracted using free sample Oragene kits and shipped to the Beijing Genomics Institute (BGI) at Shenzhen, China, for sequencing. With very limited financial resources and the simple proposition of sharing personal genomics data widely on the Internet, different sets of raw data and results were posted through blogging and social networks, describing the shared data, and reporting how results affected the family [12].

All the personal genome data were made available via figShare [1] with a public domain license (CC0), which means that companies and scientists can use the data without having to acknowledge or return any results to the family. This decision to put the personal genomics data in the public domain sped the process of adoption and collection of data by third parties, motivating several companies and scientists to contribute to the analysis. The companies included Oxford Gene Technology, InSilico Genomics, Diploid Genomics, BIOBASE, Orion Integrated Biosciences, and GeneTalk. Qiagen Ingenuity variant analysis was performed by a contributing scientist. Scientists or students who joined the crowdsourcing project at different stages added the remaining contributions. The combined crowdsourcing analyses have allowed the family to compile results from many different genome and metagenome tools.

The exomes have been used in a variety of tutorials, some of them in the USA, e.g., National Institute of Allergy and Infectious Disease, and some others in Europe, such as the Clinical Genomics module at the University of Navarra’s, MSc course in Biomedical Research taught by M.C. or an exome analysis module developed by P.N.R. for the bioinformatics curriculum at the Free University of Berlin. Some of the results from research assignment in taught courses have been fed back to the family, constituting new research additions (e.g., section on hair colour by N.T.).

**Fig. 1:**
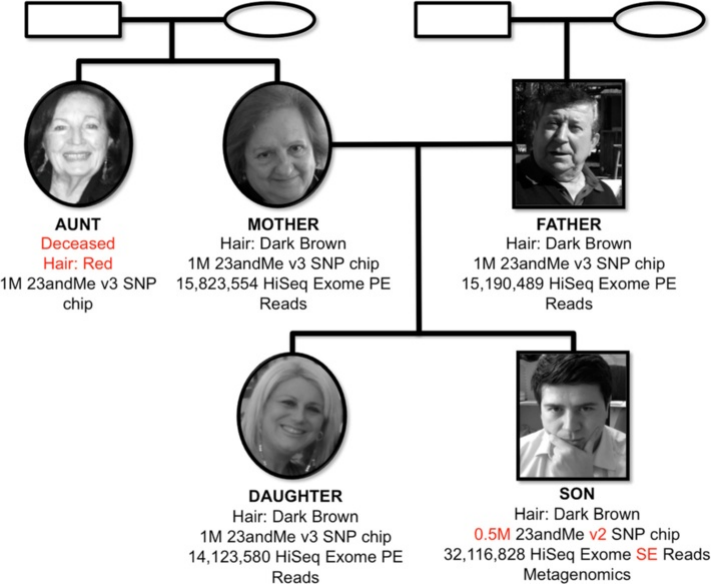
Family tree of the five individuals and the DTC tests carried out using crowdfunding and private funds. Females are represented as circles and males as squares. The genealogic tree represents the relations among the family members

In this article we present the experiences, results and a discussion of this whole process, which has been ongoing since 2009. In a previous publication [5] we presented findings and results derived from the analysis of the 23andMe genotypes from five members of the Corpas family using only SNP chip data as well as one exome and comparing extracted SNPs with SNPedia annotations. We now extend the study and analysis to the personal exomes of four members of the family and a metagenome of one of them (sadly the missing fifth exome was not to be performed due to the demise of a family member). We call members of this family Mother, Father, Aunt, Daughter and Son (Fig. 1). We contextualise the exome sequence analysis for the quartet comparing 23andMe SNP chip data with exome-derived variations, extending SNP chip data analyses with new methodologies to provide the most complete possible overview of genotype-phenotype associations making use of all crowdsourced results to date. In addition, we also delve into the ethical hurdles and barriers encountered by the family and the lead scientist of this work, who is also a member of the family.

## Results

### Comparison of exome and 23andMe data

**Fig. 2:**
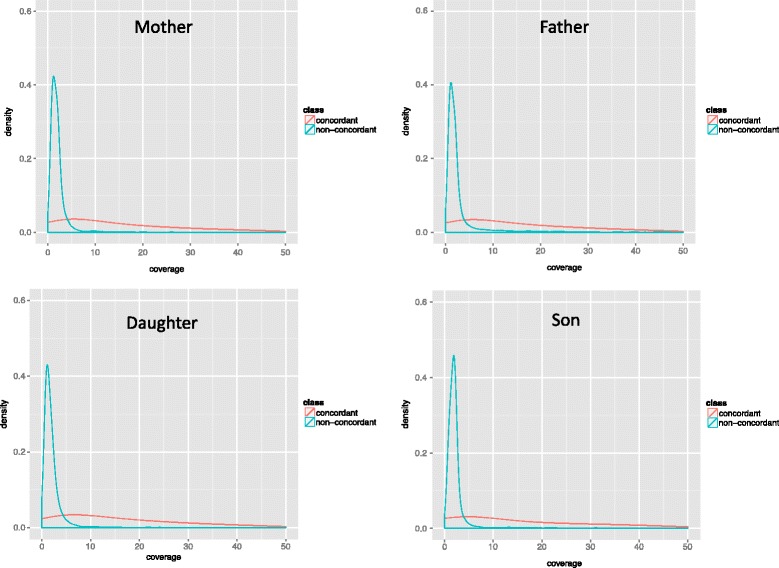
Distribution of coverage for all non-concordant SNPs between exome calls and 23andMe chips

In order to assess the quality of the data, we first compared the variant calls from the exomes to those of the previously analysed 23andMe SNPs from the same family members. SNPs were matched by ID (not by coordinates) in both the VCF exome and the 23andMe files. The scripts and data used are available via figShare. We found that exome variant calls and 23andMe SNP chips share 36,315 SNPs for Daughter, 39,624 for Father, 40,234 for Mother and 22,233 for Son. Son was genotyped using the 23andMe SNP chip version 2 and the remaining family members with version 3, hence Son has substantially fewer variants common to the exome and SNP results than the other family members. Next, we analysed the SNP calling quality for the exomes. We found that most ‘non-concordant’ SNPs (23andMe SNPs not present in the exome) have low coverage (under 10 fold). Figure 2 shows the distribution graphs for coverage in the quartet.

Among the filtered non-concordant SNPs, opposite strand SNPs and haploid predictions were included. For most errors it was not possible to establish whether the SNP calls by 23andMe or by exome analysis were erroneous. In some cases, no clear Mendelian inheritance error was found because both inferred genotypes could result in the observed family tree. For instance, rs11580218 in Father's exome genotype was GA and in 23andMe it was GT. According to dbSNP [13], rs11580218 has the alleles A/G/T.

### Hair colour in the Corpas Family

**Fig. 3:**
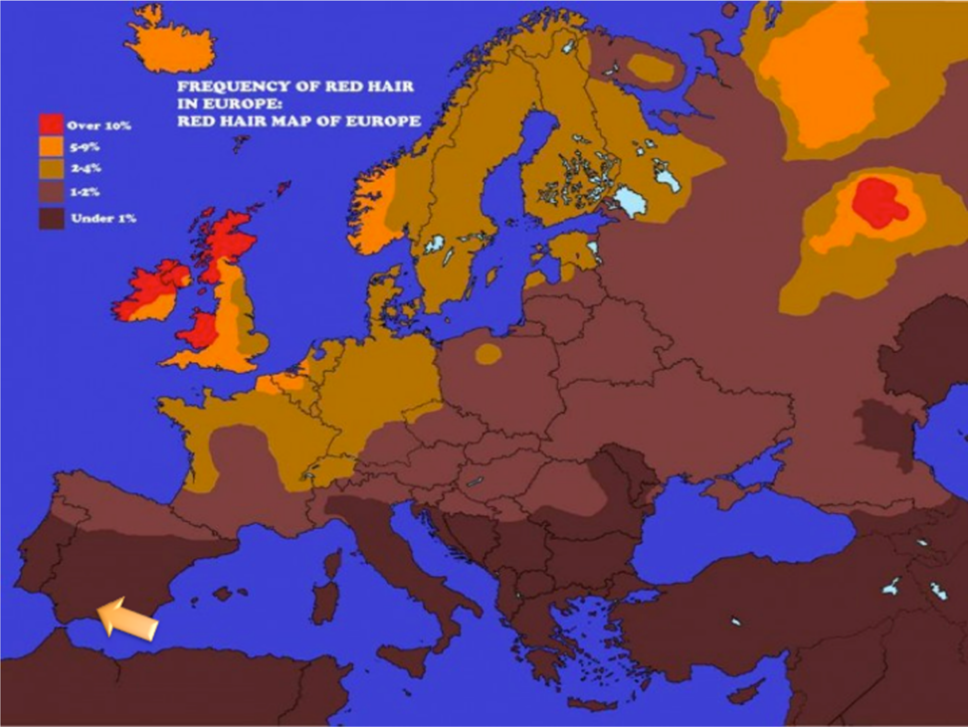
Distribution of red hair among contemporary European populations. The arrow (*bottom left*) indicates the origin of the Corpas family. [Image: James McInerney; public domain]

The main reason for focusing on the hair colour genetics of the family is because Son has three offspring, all of them with red hair, despite his black hair colour. Therefore, the objective of this section is to explain hair colour in the Corpas family using different SNP variants and comparing the genotypes of the family with other European populations. SNP data for this analysis were collected from the 23andMe SNP chips for 5 members of the Corpas family (including aunt). Figure 3 shows the distribution of red hair in Europe and the region where the Corpas family is originally from (bottom left orange arrow).

**Fig. 4:**
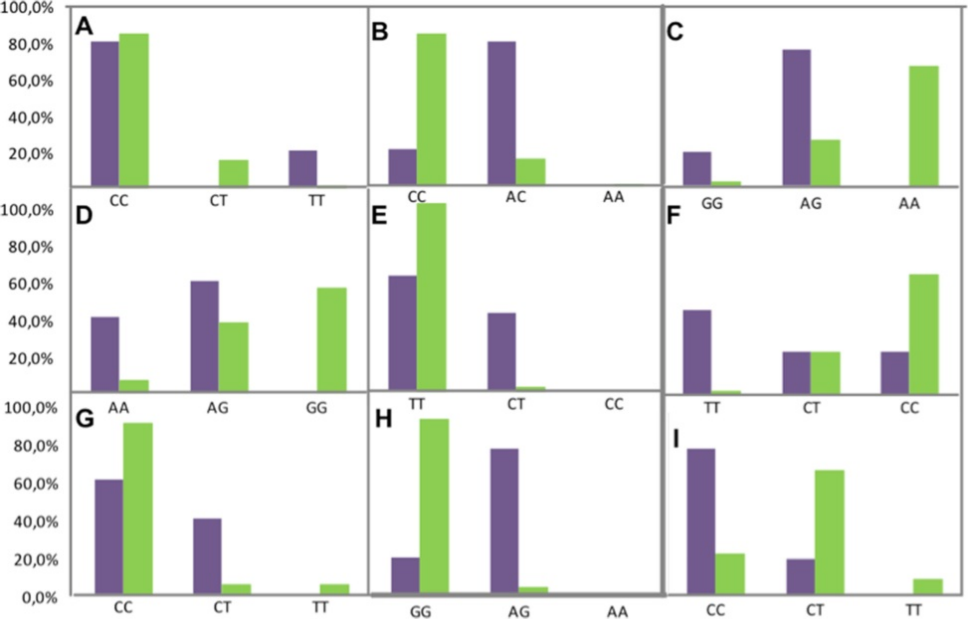
Genotype frequency of SNPs with significant differences between the Corpas family (*purple*) and a Polish reference population (*green*). See Methods for details on how the reference population was obtained. **a** rs122203592; **b** rs7183877; **c** rs2402130; **d** rs12913832; **e** rs11635884; **f** rs916977; **g** rs1800407; **h** rs11547464; **i** rs1015362

We characterised 27 SNPs from 10 genes associated with hair colour from the Corpas family: SLC45A2 (rs16891982, rs28777); IRF4 (rs12203592); TYRP1 (rs2733832, rs683); TPCN2 (rs35264875, rs3829241); TYR (rs1042602, rs1393350); SLC24A4 (rs4904868, rs2402130); OCA2 (rs1800407, rs7495174); HERC2 (rs12913832, rs7183877, rs11635884 rs916977, rs8039195); MC1R (rs1805005, rs1805006, rs2228479, rs11547464, rs1110400, rs1805008, rs885479) and ASIP (rs1015362, rs2378249). Significant differences (*p* <0.05) in the genotype distribution were sought. Genotypes with significant differences between the Corpas family and a reference population are shown in Fig. 4.

**Fig. 5:**
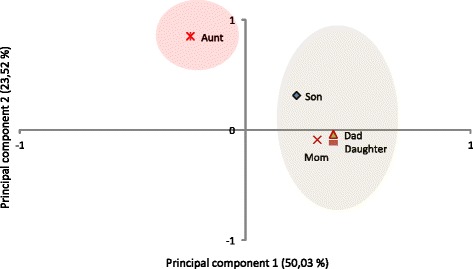
Score plots generated from the principal component analysis of Corpas family genotypes for hair colour. *Red* and *grey* shaded areas show red and dark-brown hair respectively

Principal Component Analysis (PCA) was performed to analyse the distribution of variants in members of the Corpas family. This analysis showed a clear difference between the family member with red hair (Aunt) and those with dark brown hair (Fig. 5). This difference could be explained due to variants in the MC1R gene [14, 15]. In addition, Aunt was found to have a different genotype for rs916977, rs8039195 and rs7183877 in the HER2 gene.

Both populations (Corpas family and reference) have similar genotype pattern and significant differences are only present in variants of 9 SNPs. According to previous studies [16], genotype of hair colour SNP variants are different between the two European populations. Nevertheless, few differences have been found between these two cohorts, which may be due to the Polish population of the reference study having red hair preferably [17] and red hair being present in the Corpas family. In addition, we have been able to successfully conclude that Son is indeed a red hair carrier, explaining why all his offspring has red hair.

### Analysis of exomes from four different platforms

**Fig. 6:**
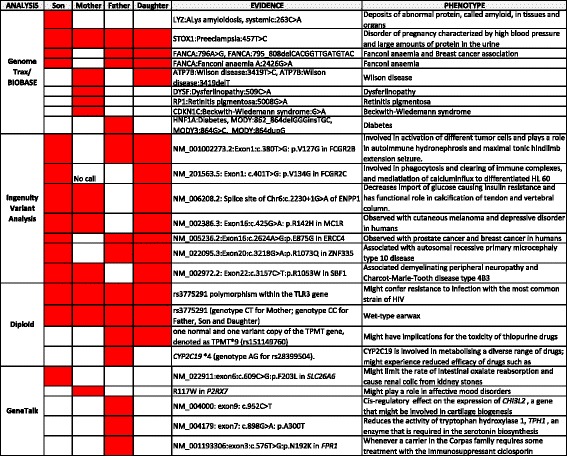
Comparison of most significant exome results from the crowdsourced analyses of the Corpas family quartet by four different platforms: Genome Trax, Ingenuity Variant Analysis, Diploid and GeneTalk. The different predicted phenotypes and their evidence are represented as present (*red highlight*) or absent (*blank*) for each of the family individuals. We find that there is no overlap among reported top results from the four companies

The VCF file for the exome quartet was analysed using a crowdsourced approach. Figure 6 displays the summary of main findings for associations between observed variants (evidence) and their predicted phenotype. Each of the methods uses different pipelines offered by four different platforms, including Genome Trax of BIOBASE (Qiagen, Beverly, MA, USA), Ingenuity Variant Analysis (Qiagen, Redwood City, CA, USA), Diploid genome interpretation service (Diploid Genomics, Leuven, Belgium), and GeneTalk (GeneTalk, Berlin, Germany). One of the main observations from this combined analysis is that each platform provides a substantially different set of results.

Within the different analyses, there are common trends for most members of the family. For example, Genome Trax predicted that all members of the family are susceptible to preeclampsia. It also predicted a deleterious mutation in the Fanconi Anaemia FANCA gene in all quartet members except Mother. The Ingenuity variant analysis, based on a dominant genetic model, predicted pathogenic variants in ectonucleotide pyrophosphatase/phosphodiesterase 1(ENPP1) and a likely pathogenic variant for the melanocortin 1 receptor (MC1R) in the three family members except Father, associated with red hair and non-tanning skin.

Diploid reported two main findings for all family members: a) they all carry a variant that has been associated with an increased resistance to the common HIV strain infection and b) they all are predicted to have wet type earwax. GeneTalk did not predict any trait that is common to all members of the family. For Son, it predicted a greater risk of renal colic from kidney stones. Our Additional files 1, 2, 3 and 4 document provides the complete reports from analysis platforms. On the whole, the results have not revealed any clear genetic risk factors that would necessitate substantial changes in lifestyle or medical management.

### Interpretation and relevance

An important question for DTC genomics analysis is whether consumers will be able to interpret the findings without genetic counselling or other medical advice. One of the authors (P.N.R.) is active in the field of medical genetics, and has attempted to assess the understanding of the family before and after discussions with Son about the findings. Son (Manuel Corpas), who is himself a scientist and bioinformatician, has no medical background but is still likely to be much better able to interpret the relevance of the findings than a member of the general public without either medical, bioinformatics, or scientific training. We have held several discussions about the reports of the four DTC providers. Perhaps one of the most striking observations about the reports is the sheer number of findings predicted to be deleterious. A typical exome displays tens of thousands of variants compared with the genomic reference sequence, up to roughly 10,000 of which are predicted to lead to nonsynonymous amino acid substitutions, alterations of conserved splice site residues, or small insertions or deletions. Even after filtering out common variants, hundreds of variants of suspected medical relevance may remain. In fact, each genome is thought to harbour about 100 genuine loss-of-function variants with about 20 genes completely inactivated [18].

Son reported that the list of potentially relevant variants conveyed by the DTC providers were simply too copious to evaluate in detail in discussions within his family. Genome Trax reported 162 variants in candidate genes as deleterious. These included homozygous variants calls in genes related to cancer (BRCA1, APC, MEN1), congenital malformation syndromes (TCOF1), metabolic deficiencies (FBP1, HEXA), and neurological diseases (FUS, MLC1, DMD). “Major variants”, included the LYZ gene that was homozygous in Son and heterozygous in each parent. Mutations in this gene are associated with familial visceral amyloidosis (OMIM: 105200), a disease characterized by chronic nephropathy, arterial hypertension, and hepatosplenomegaly. The report notes that the allele is rare in the general population and is predicted deleterious by a bioinformatics tool. The variant in question is listed in dbSNP with ID rs1800973, which is reported to have a minor allele frequency of 0.021. Since familial visceral amyloidosis has an autosomal dominant mode of inheritance, and none of the family members show signs of this serious disease, the variant can confidently be classified as non-pathogenic.

Another variant flagged by the Genome Trax analysis involved the P2RX5 gene. It is noted that each parent is heterozygous for a frameshift deletion, but the Son is homozygous wild type. The variant was highlighted because it is associated with “allogenic cytotoxic T lymphocyte response”. However, the publication that reports this finding was referring to an allogeneic cytotoxic T lymphocyte response in a patient with chronic myeloid leukemia after donor lymphocyte infusion. This patient was found to have the frameshift mutation in homozygous state [19]. Since parents are heterozygous, and Son is homozygous wild type at this position, and since no family member has been diagnosed with chronic myeloid leukemia, from a medical standpoint this finding is irrelevant.

The variant c.2562T > G was reported to cause altered splicing in CFTR, the cystic fibrosis gene. Mother is reported heterozygous for the variant, and Son is reported as being homozygous for the wild type, with the interpretation that this is just a disease-associated polymorphism. The variant was initially reported to be associated with increased CFTR exon skipping in healthy individuals and was thought to disrupt an exonic splicing enhancer, although no experimental proof was provided for this assertion. The variant is listed in dbSNP under rs1042077 with a minor allele frequency of 44 %, and is interpreted as “Benign/Likely Benign” in ClinVar [20]. Medical interpretation would classify this as irrelevant, again especially in light of cystic fibrosis being an autosomal recessive disease and the lack of any convincing evidence of pathogenicity.

A predicted pathogenic variant in the gene STOX1 was found to be homozygous in Son. Some mutations in this gene are associated with preeclampsia, which is noted to be a condition only relevant in pregnancy and thus not applicable to Son. While this interpretation is reasonable, the fact that this variant (rs1341667) has a population minor allele frequency of 36.7 %, makes it unlikely that it is a large-effect mutation that would be a major risk factor.

The Ingenuity Variant Analysis software predicted a number of single nucleotide variants as pathogenic (Fig. 6). For instance, NM_006208.2:c.2230 + 1G > A in the ENPP1 gene has been associated with metabolic syndrome in obese childhood [21, 22] and is observed in Mother, Son and Daughter. A variant in MC1R (NM_002386.3:c.425G > A, p.R142H) has also been predicted to be likely pathogenic, was observed in Mother, Son and Daughter, and has been reported to be associated with increased risk of developing melanoma [23]. In fact Aunt (analysed using 23andMe data) sadly passed away in 2013 as a consequence of malignant melanoma [1]. Further, a variant in the SET binding factor 1 (SBF1) gene (NM_002972.2:c.3157C > T, p.R1053W) is observed in all samples except Father. SBF1 is associated with demyelinating peripheral neuropathy and Charcot-Marie-Tooth disease type 4B3 (OMIM: 615284). Certain variants that occur in Father, Son and Daughter (not Mother) are in the excision repair cross-complementation group 4 (ERCC4) gene and the zinc finger protein 335 (ZNF335). A mutation in the ERCC4 gene can be observed with prostate cancer [24] and breast cancer [25].

The report from GeneTalk was prepared by the coordinator of the project, Peter Krawitz, who is a medical doctor. GeneTalk provides a consideration of variants in a number of genes and rules out medical relevance for almost all of them, but does discuss the possibility that a de novo variant in SLC26A6, which codes for an anion exchanger, might limit the rate of intestinal oxalate reabsorption [26]. This is a risk factor for oxalate kidney stones and renal colic, even though mutations in this gene have not previously been found to be causative of human disease. It should also be noted that the evaluation of Peter Krawitz was part of the crowdsourcing project described here, but that users of GeneTalk by default do not receive a medical evaluation of the variants.

The exome analysis by Diploid Genomics, yet again, emphasised a different set of variants. The heterozygous presence of a variant of the TPMT gene (denoted as TPMT*9) was detected in both Father and Daughter. Given this is an extremely rare allele, its exact functional status and pharmacological implications are still preliminary, but carrying this allele has been suggested to have possible implications for thiopurine toxicity [27–29]. Father was shown to carry one normal and one defective allele of the CYP2C19 gene, denoted as CYP2C19*4. This intermediate metaboliser status can impact the efficacy of drugs metabolised by the CYP2C19 enzyme, such as clopidogrel.

### Analysis of son’s metagenome

**Fig. 7:**
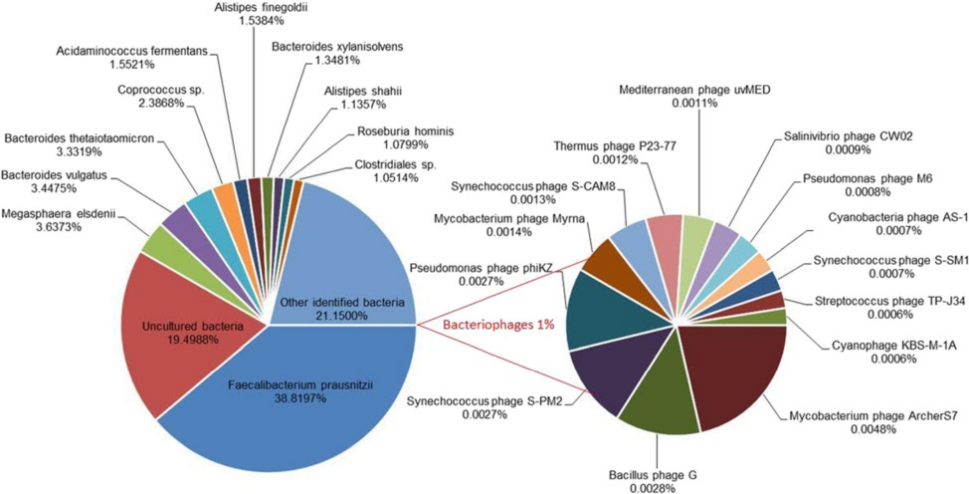
Metagenomics analysis of DNA in faeces of Son. The taxonomic composition based on DNA matching genomic signatures and motif fingerprints of different bacterial and phage genomes is shown

To date only one individual from the Corpas family has undergone metagenome sequencing. Hence here we report the crowdsourced analysis from this individual only. Taxonomical composition analysis of more than 2 million 100 nucleotide reads was performed using a library of microbial genomic signatures and motif fingerprints based on [30, 31]. Figure 7 shows the percentage of microbial species for whose DNA is present in the sample. Approximately 39 % of the DNA belonged to genomic signatures of the species Faecalibacterium prausnitzii, excluding all known strains, 18 % to uncultured bacteria and rest to ~1000 bacterial species belonging to the Bacteroides and Firmicutes phyla. These include the genera Megasphaera spp., Bacteroides spp., Coprococcus spp., and Clostridiales spp. Less than 1 % of DNA was mapped to lytic bacteriophages, members of genera I3likevirus, T4likevirus and Phikzlikevirus of the family Myoviridae. No motif fingerprints corresponding to known viruses infecting eukaryotic cells were detected.

## Discussion

We have presented the results of the crowdsourcing of the analysis of a genomic dataset for a family. The current lack of whole-family personal genomics data available for public use has been a significant factor in the success of this family’s crowdsourcing enterprise, attracting the attention of scientists and companies to contribute results. We have contributions from scientists who wanted to carry out an experiment to analyse a cohort of individuals or test data for development of new tools (SNPedia tools [7], Ingenuity analyses) or for demonstrating new products to prospective customers and clients (InSilicoDB, BIOBASE Genome Trax, Orion Integrated Biosciences Inc., GeneTalk, and Diploid Genome Interpretation).

The main goal of this family study has been to explore the utility of DTC genomic analysis in a family setting with a data derived from personal genotypes, exomes and metagenomes. In order to achieve this objective, many obstacles had to be dealt with (e.g., finding a sequencing provider, shipping samples, establishing the appropriate ethical framework to perform and publish the analyses). We expect this study to pave the way for other families who might wish to actively engage in participatory medicine and share their experiences.

Some individuals have already made their genome sequences public (e.g., Personal Genome Project) and many more individuals have done so with their 23andMe data (e.g., Genomes Unzipped). The Corpas family decided to take the unprecedented move of publishing their exome and microbiome data and analyses on the Internet under a CC0 license waiver, the least restrictive type of license. To our knowledge, this is the first reported family to engage independently in genomic citizen science by publishing their identities and personal genomes on the Internet. This does not mean that the family encourages everybody to follow this example; the family does request, however, other researchers and the general public not to censure this decision. This has allowed them to explore their personal genomes to an extent it would have been impossible at this time otherwise.

It has been argued that individuals have more control due to participatory nature of research enabled by having access to personal genomic data. However, it might be also the case that by letting individuals share their data and accept more responsibility about potential consequences, they lose their control on their data and results they might receive. In addition, posting data in such an identified way would make personal genomic data sharing an irreversible decision in nature, given the fact that withdrawing yet shared data from the web is not a feasible option.

In order to satisfy established ethical conventions by the community, ethical research and advice were sought to create the appropriate framework in which to share and report data and findings. Although the Internet and private test providers rendered the personal genomic data sharing a plausible practice, it is hard to perceive that this is immediately translated to more power to individuals. There are significant limitations on controllability of data which has to be taken into consideration while promoting participant-centric approaches to research. Participant centric research and in this case personal data sharing is often coupled with presuming a higher level of responsibility and awareness for the participants. Publicly sharing data will put third parties’ access to data under no limit whatsoever. As a result, third party researchers are not obliged to sign any contract or abide by “terms and conditions” that normally set in other research settings. This might raise concerns that in case of any harmful uses by third party researchers, there are no grounds for making researchers legally responsible. To this end, it is imperative to assure those who engage in such initiatives are sufficiently informed about implications and prepared to embrace the associated risks. It is not the objective of this research to ignore current practice and established ethical procedures in the data sharing of personal genomes (see NIH’s elements of informed consent [32] and the Global Alliance for Genomics and Health [33]).

A primary objective was to probe the extent to which DTC genetic testing can provide meaningful results to people who decide to purchase those tests privately. By lowering the barriers for scientists and companies to access these data, the family could increase the chances of successfully crowdsourcing their analysis and receiving results back. These data have been used for testing and educational purposes.

The health care implications of the exome and metagenome data obtained in our study are at best uncertain. It was remarkable that there was no overlap of the sets of variants reported as significant by the four DTC analysis providers. This is reminiscent of recent reports on the low concordance of DTC analysis of SNP-based testing, with substantial differences in the predicted disease risks [34–36]. The lack of concordance between the results of the DTC analyses of the Family’s exomes illustrates the difficulties in using this kind of analysis for participatory medicine at present, and it is not clear if any of the findings have relevance for the clinical care of the family at present. A further limitation of our study is the fact that for economic reasons, whole exome sequencing was performed at a relatively low average coverage of about 30×. It has been noted that different bioinformatic pipelines lead to different sets of called variants [37]. We were limited to one pipeline provided online by InSilicoDB [9], and did not explore the effects of different computational analysis strategies, since this would not normally be available in a DTC context.

We note that our results were not intended to compare the ability of the DTC companies to identify disease-causing mutations in the setting of human genetics or oncology, which are the primary use cases for exome sequencing. We provide the same starting point, a VCF file, and the same starting end, the predicted phenotypes based on SNP variations from the VCF file. The analyses that were conducted at a particular point in time will have their own software versions and configurations. It is thus almost impossible to have a 100 % reproducibility of results due to the difficulty posed by the various formats presented from the different providers and the dynamics of the evolution of methods or the businesses themselves.

The metagenomic analysis of Son’s fecal microbiome did not identify any actionable findings. The most abundant bacterium in the sample, Faecalibacterium prausnitzii, is thought to be protective [38], but there is currently no accepted analysis strategy that would allow one to make certain conclusions about the medical relevance of the results.

## Conclusion

In this study we have confined ourselves to the evidence given by DTC companies and applied different software for interpretation. One of our main aims was to simulate what it will be for a family of the future to analyse their personal genomes privately. At current costs, however, this type of analysis could hardly be afforded by most families globally as genetic testing and counselling would have been too expensive. This may change in the future as the cost of analysis drops.

We have shown the extent to which private efforts in participatory medicine can enhance knowledge or a family of committed members wishing to analyse their personal genomes. We have also shown how current technologies allow entire families to engage in participatory research with the help of the Internet without having to consult a clinician beforehand. While carefully pondering risks and seeking appropriate ethical framework, the Corpas family decided to publish all their genetic data on the Internet to crowdsource their analysis. It is our belief that the risks of unintended uses of this family’s genomic data have far been outweighed by the amount of information received from the contributors to the crowdsourcing of data analysis. The results of the analysis provided an explanation for the “surprising” hair colour of Son’s children, and indicated the possibility that Father may be an intermediate metaboliser of the antiplatelet medication clopidogrel (if there were an indication for this medication, an alternate medication such as prasugrel might be considered).

Combining the results of four providers, we have offered an initial survey into the amount of knowledge that can be gathered by a family using solely DTC means. Results from the DTC providers vary widely between them and no consensus seems to come up in terms of actionable knowledge derived from the analyses. In spite of the limited amount of information gained, this study offers an initial survey on what is possible for any ordinary family to learn about their own personal exomes with current sequencing technology.

## Methods

### Ethical framework

Since the family bought the sequencing tests themselves, they were not required to follow an Institutional Review Board (IRB). We have, however, consulted with ethical and genetic counselling experts regarding the appropriate way in which to carry out the public sharing of personal genomic data, which led us to adopt the consent forms developed by Genomes Unzipped [39]. Genomes Unzipped developed a participant information form clarifying the goals of the personal genomics data sharing partakers were involved in. We adapted this participant form and had it signed for all members of the family quartet, clarifying the goals of the personal genomic data sharing, potential risks and discomforts derived from the (ab)use of the data shared on the Internet. This consent process was discussed and agreed upon by the Corpas family. Issues discussed included that, subsequent to publicly sharing the data in the public domain, data could no longer be considered to belong exclusively to the individual; therefore, it would be hard to prevent unintended usages in the future, if not impossible. Additionally, it was also indicated that from the legal stand point of view (depending on jurisdictions) the family could relinquish all rights over the data by publicly sharing them. From the standpoint of genetic counselling, it was also apparent from our expert consultant that it is difficult to compare our study to a 'gold standard' regulatory practice because there is not such a thing yet. Hence we are unable to assess the significance of the results in relation to current practice.

### Crowdsourced data

From 2011 onwards, the Corpas family decided to embark on the journey of analysing their personal genomes. When the first exome was obtained (2012), Next Generation Sequencing (NGS) was becoming routine in research labs but was rarely available for DTC personal genomic tests. Son was in fact the first DTC personal genome that was sequenced by the BGI. After this, some negotiations followed, with BGI agreeing to perform Illumina Whole Genome Sequencing (WGS) for $4,000 per person (2012 prices). To raise funds, a crowdfunding campaign was started to fund $20,000 for five genomes [40]. The crowdfunding plea offered in return (for a significant percentage of the money expected to be raised) early access to the data and printouts of publications derived from the analysis of the data. To our knowledge this was the first time crowdsourcing and crowdfunding were employed for the analysis of the genomes of a complete family. The crowdfunding campaign succeeded in raising ~ $3,300, precluding whole genome sequencing of the entire family; hence exome sequencing for 3 members of the family was performed, a much cheaper alternative. The crowdfunding initiative, together with private efforts from the family, provided sufficient funds to generate a variety of datasets (Fig. 1).

The crowdsourced data include both raw and processed data. Because these data were obtained as DTC tests, sometimes the source or platform utilised for the same test was slightly different between individuals because a different version of the platform was used (indicated in Table 1). We have attempted to minimise the impact of platform versions by using the same pipelines and methods for alignment and variant calling.

**Table 1:** Summary of the crowdsourced data with its metadata. The dataset includes a total of 27 files and 18,463 MB of data

Type	Provider	Source	Platform	Format	Year obtained	# files	Individuals(s)	Approx. size (MB)
Genotype	23andMe	Saliva	SNP chip v2	bed.zip	2009	1	Son	5
Genotype	23andMe	Saliva	SNP chip v3	bed.zip	2011	1	Mother	8
Genotype	23andMe	Saliva	SNP chip v3	bed.zip	2011	1	Father	8
Genotype	23andMe	Saliva	SNP chip v3	bed.zip	2011	1	Daughter	8
Genotype	23andMe	Saliva	SNP chip v3	bed.zip	2011	1	Aunt	8
Annotation	SNPedia	SNP chip v2	Phenotypes	txt	2012	1	Son	0.3
Annotation	SNPedia	SNP chip v3	Phenotypes	txt	2012	1	Mother	0.4
Annotation	SNPedia	SNP chip v3	Phenotypes	txt	2012	1	Father	0.4
Annotation	SNPedia	SNP chip v3	Phenotypes	txt	2012	1	Daughter	0.4
Annotation	SNPedia	SNP chip v3	Phenotypes	txt	2012	1	Aunt	0.4
Whole Exome Sequencing	BGI	Saliva	SE Illumina HiSeq 2000	fastq.gz	2011	4	Son	2400
Whole Exome Sequencing	BGI	Saliva	PE Illumina HiSeq 2000	fastq.gz	2013	2	Mother	2000
Whole Exome Sequencing	BGI	Saliva	PE Illumina HiSeq 2000	fastq.gz	2013	2	Father	2000
Whole Exome Sequencing	BGI	Saliva	PE Illumina HiSeq 2000	fastq.gz	2013	2	Sister	2400
Alignment	InSilicoDB	SE Illumina HiSeq 2000	BWA	bam	2013	1	Son	2900
Alignment	InSilicoDB	PE Illumina HiSeq 2000	BWA	bam	2013	1	Mother	1800
Alignment	InSilicoDB	PE Illumina HiSeq 2000	BWA	bam	2013	1	Father	1600
Alignment	InSilicoDB	PE Illumina HiSeq 2000	BWA	bam	2013	1	Daughter	2200
Annotation	InSilicoDB	Bam from Son, Mother, Father, Daughter	GATK	VCF	2013	1	Son, Mother, Father, Daughter	124.6
Metagenomics	BGI	Fecal frozen sample	PE Illumina HiSeq 2000	fastq.gz	2013	2	Son	1000
Total						27		18463.5

The crowdsourced data encompass genotypes, annotations, alignments, whole exome sequencing and metagenomics sequencing. The fastq files, the quartet’s VCF file and their corresponding metadata are available via figShare. InSilicoDB [9] was used to create the BAM and VCF files. From InSilicoDB it can be downloaded the fastq files, BAM files and VCF files for each member of the family quartet [41–44]. Although the genotype data and derived processed sources have been published elsewhere [5], the exome sequencing data, and the corresponding derived (processed) files and metagenomics data are presented for the first time here. All variations are single nucleotide variants (SNVs) and no indels or copy number variations were used for this analysis.

The years in which the data were obtained are denoted in Table 1 to roughly show the timeline of family tests. The five sample genotype data obtained from 23andMe SNP chips are in bed format, mapped to the human reference genome, NCBI36. These datasets were obtained in the summer of 2009 (Son; version 2 of 23andMe; ~0.5 M SNPs) and January 2011 (Mother, Father, Daughter, Aunt; 23andMe SNP chip version 3, ~1 M SNPs per individual). Whole exome sequencing from Son, Mother, Father, and Sister was performed with DNA extracted from saliva using Illumina HiSeq 2000 with an average coverage of the target region of ~30×. The Son’s exome was the first family member to be sequenced (January 2012). Agilent SureSelect Target Enrichment technology (human all exon V4) for single-end reads was used to capture DNA material. The other three exomes (Mother, Father, and Daughter) were sequenced in January 2013 using the Agilent SureSelect 44 Mb human All-Exon design and sequenced using Illumina HiSeq. All sequencing was done by BGI. The metagenomics sequencing was performed for Son in August 2013 from a fecal sample using Illumina (HiSeq 2000) shotgun sequencing. We crowdsourced only the resulting two cleaned fastq PE metagenomics files. These contain an insert size of 170 bp and a total size of 1,201 Mb of clean data (after removal of carrier reads, low complexity reads, low quality reads, adapter contamination and duplicate reads).

### Crowdsourcing the analysis

All of the crowdsourced contributors are included as authors or defined as providers. Please see the “Author Contributions section” or Table 1 for more details. The policy adopted by the family was of complete transparency and of sharing all available data on the Internet as soon as it was possible. Releasing all data under a Creative Commons License 0 waiver effectively allowed any possible use by third parties to the extent permitted by law. After an initial period in which all data were made available through the *Manuel Corpas’ Blog* [12], it soon became apparent that this venue would make it increasingly difficult to provide a centralised place with all available data sources. A solution was found through figShare, a resource that allows upload of data from experiments, giving them a digital object identifier, which makes them citable. figShare is also useful for tracking the number of downloads, views and shares via Facebook and Twitter. All data available for the family can be accessed via figShare under the project tag ‘*Corpasome*’. To date, the Corpas Family Trio Exome dataset (Mother, Father, Daughter) in figShare has received 1247 views and 2 shares.

Following the posting of the family genome data (SNP, exome and microbiome), notifications and contributions have been received from users who found them useful for their tool development or experiments. It therefore became apparent how valuable these data were for many scientists or companies developing or testing their products. This unexpected engagement by the community can be mostly explained by the scarcity of publicly available familial data with an unrestricted license like CC0 on the Internet. Arguably this has worked to the advantage of the family’s goal to crowdsource the analysis of the *Corpasome*. Since the data were posted, results have been received through a variety of channels including comments to blog posts, direct emails, Twitter or Facebook messages. Previous findings with the family using mainly 23andMe data have been published elsewhere [5]; results involving the analysis of NGS data integrating four exomes and a microbiome and how they compare to 23andMe data are reported here for the first time.

### Red hair analysis

Reference data were obtained from two different sources. First, Polish population data were obtained from a study of Branicki et al. [17] who genotyped 385 samples of unrelated Europeans living in Poland and genotyped a subset of 25 SNPs via mass spectrometry using Sequenom multiplexing. The second reference population refers to Nurses’ Health Study cohort from Han et al. [14] research, including women with European ancestry, who were genotyped using the Illumina Human-Hap550 array. Genotypes from these two reference populations were deduced applying Hardy-Weinberg equilibrium rules using the known Minor Allele Frequency. Allele and genotype frequencies were computed assuming that both populations are in Hardy-Weinberg equilibrium. All the statistical analyses were performed using SPSS Statistics (IBM, New York, USA), Version 21 for Windows. In order to compare two populations and discriminate if they have different hair colour information, a goodness of fit test was conducted. The goodness of fit test reported a contingency table that could be used to study genotypic frequency differences between “family” and “reference” cohorts. The *χ*^2^ and Fisher´s exact statistic were calculated. In order to infer statistical significance, only the Fisher exact test was used due to the small size of Corpas family.

### Exome data analysis

Processing of exome data was performed using InSilicoDB [9] a web-based storage hub. For this dataset we used the InSilicoDB pipeline v0.8. For all exomes, InSilicoDB produced bam files with BWA [45]. This comprised the alignment of an average 27,181,028 paired end reads per sample to reference genome GRCh37/hg19. We removed duplicate reads using Picard MarkDuplicates (v 1.70) [46], performed local realignment around indels, base quality score recalibration and variant calling with the Genome Analysis Toolkit (GATK v 2.1.13) ‘best practice variant detection method’ pipeline [1, 47]. We are aware that variant calling using GATK version 2.1–13 has had known bugs causing loss of function false positives. Variant discovery was performed by InSilicoDB using joint variant calling, outputting one VCF file for all four processed exomes. Variant calling is executed independently for each sample, followed by joint genotyping. No filtering was performed at this stage. Quality values computed along the pipelines were passed to the output VCF file and used in the next stage. The VCF file was used to perform the analysis using four types of software by different company providers.

1. **Genome Trax:** Filtered non-HGMD common variants (>1 % allele frequency in dbSNP) [48]. Annotations were added from HGMD [48], the GWAS Catalogue [49] and ClinVar [22]. Allele frequencies for EVS and the 1000 Genomes [50] pilot for the CEU population were provided. Also, included nonsynonymous SNP functional predictions of dbNSFP and Orphanet Inheritance [51]. Effects such as hom-from-het, *de novo* or compound-het variants have also been filtered. Variants not passing the quality filter were discarded. Variants were then ranked according to the rules of being a known disease causing mutation and being observed in a homozygous manner, or observed in a heterozygous manner but the variants for the associated gene were reported to be dominant. Also, considered as additional evidence (though, not used for ranking): rare variant’s allele in the background population (in cases where such numbers are known), variant predicted to be deleterious by several prediction tools and an exact match of the variant for the nucleotide change (Additional file 2).
2. **GeneTalk:** Various filter settings were implemented for analysis using GeneTalk [52]. First, variants of Father and Mother were analysed to look for screening of pathogenic heterozygous risk alleles that might cause severe recessive disorders discussed in the scientific literature. The following filters were applied: a) functional filter (missense, nonsense, splicing and frameshift); b) frequency filter (minor allele frequency <1 %); c) Inheritance filter (heterozygous genotypes only); d) annotation filter (rated by GeneTalk community at least suspicious in more than 1 individual. Second, the exome quartet was then analysed assuming Son was affected by some trait. We filtered for possibly pathogenic homozygous variants that might cause recessive diseases. Third, de novo mutations in the son were filtered, removing all dbSNP entries, setting the Son’s status to affected and filtering for dominant.
3. **Diploid genomics:** Variants were filtered based on quality, frequency, genomic position, protein effect, pathogenicity and previous phenotypic associations. For each selected SNP, location, genotype, sequencing and allele depth, genotype quality, Phred likelihood, SIFT [53] and PolyPhen-2 [54] scores were provided if available. The selected SNPs were ranked according to their predicted deleterious effect on the encoded protein. Homozygous calls were also identified. SNPs for which a genotype-phenotype association is described in scientific literature, and for which no conflicting results have been published, were reported. In addition, the potential clinical relevance of novel variants with a predicted damaging effect (based on *in silico* prediction tools and type of mutation) was also assessed. Risk factors for common complex disorders were not part of the analysis. All called SNPs that are not included in dbSNP were selected from the VCF files (e.g., 22859 SNPs for Son). Of those, we filtered all SNPs that are located in genes which are tested in the standard Diploid analysis (about 3000 genes) (e.g., 1495 SNPs for Son). Of this selection of SNPs, we further selected those in the coding regions of the genes (e.g., 88 SNPs for Son). For each of these SNPs, the location, genotype, sequencing and allele depth, genotype quality, Phred likelihood and SIFT and PolyPhen scores are provided if available (see Additional file 3). The selected SNPs are sorted from high to low agony, based on the SIFT and PolyPhen scores. None of the variants listed in the four probands have proven clinical significance, we can therefore not draw any conclusions for these variants at this moment. However, we sorted the variants by relevance based on the predicted deleterious effect of the mutation on the encoded protein (predicted by SIFT and PolyPhen scores with scores of >0.8 and <0.05 predicted to cause deleterious effects, respectively).
4. **Ingenuity variant analysis:** Analysis was performed on four samples (Daughter, Son, Father and Mother) by importing variant call data. All the variants were annotated using the available content from the Ingenuity Knowledge Base. The GRCh37 (hg19) human reference genome was used as standard in the application to compare with variants. A series of filters discussed below were applied to the variants (Additional file 1: Figure S1) At the top of the variant filter cascade there were 300,717 called variants across the four samples (2 cases, Daughter and Son, and 2 controls, Father and Mother). These variants were associated with a total of 17,838 genes as defined by RefSeq gene model. A confidence filter was applied to keep variants with call quality >20 for the samples and, excluded variants in the top 5 % most exonically variable 100 base windows in healthy public genomes and the top 1 % most exonically variable genes in 1000 genomes. The common variants filter excluded variants with an allele frequency of at least 3 % that were observed in the populations in 1000 Genome project, public Complete Genomics genomes and the Exome Sequencing Project of the National Heart Lung and Blood Institute. The predicted deleterious filter was enabled to retain variants that were experimentally observed to be associated with a phenotype (pathogenic and likely pathogenic), associated with gain of function (as established in the literature) or associated with loss of gene function (frameshift, in-frame indel, or start/stop codon change, and missense, not predicted to be tolerated by SIFT and PolyPhen-2). Genetic analysis filter was used to construct different inheritance models. In the ‘Dominant’ inheritance model of the Genetic Analysis filter (Additional file 1: Figure S1) the variants were restricted to ‘transmitted’ (from controls to cases), ‘heterozygous’ genotype in cases and, with genotype occurrence in 2 of the 2 case samples at variant level. In controls, other variants (homozygous, compound heterozygous, haploinsufficient, hemizygous and het-ambiguous) except ‘heterozygous’ genotype, were excluded and, with genotype occurrence in at least 1 of the 2 control samples at variant level. A second Genetic Analysis filter was applied to exclude ‘homozygous’ and ‘heterozygous’ variants in controls and, with genotype occurrence in 2 of the 2 control samples at variant level.

### Metagenomic (microbiome) data analysis

All metagenomic bioinformatics analyses were performed on Orion Integrated Bioscience servers using motif fingerprint generation (MF-gen) and CHAST algorithms to identify protein segments conserved across a specific taxonomy [30, 31]. MF-gen implementation is based on the assumption that 12-amino acid long motifs do not overlap linearly but they may be contiguous in 3-dimensional space of non-redundant protein sequences. CHAST assigned the taxonomy coverage of each motif by exhaustive protein database released by GenBank. From the scanning process three types of motif fingerprints (MF) were recognised: 1) *MF-Type I* segments specific to a given taxonomical group (e.g., species, sub-types or strain); 2) *MF-Type II* segments shared by a host and pathogen only. These might have been co-opted by the pathogen to affect immune signalling, regulatory or metabolic pathways. 3) *MF-Type III*, non-specific segments shared by more than two species. Each MF was mapped to their corresponding coding region or genomic signature. For this analysis, the *MF-type I* library for bacteria and viruses was scanned through the individual’s reads and the taxonomic composition was represented as percentage.

### Deposition of data

Exome data used is deposited in figShare [http://figshare.com/articles/Corpasome/693052] and can be freely downloaded and used under a CC0 license.

### Supporting data

Supporting data and results are available in the Additional files 1, 2, 3 and 4.

## Endnote

^1^Contrary to the evidence, Aunt’s 23andMe report predicts a decreased risk for Melanoma based on two SNPs (rs1805007 and rs1805008) located in *MC1R*.

## Additional files

Additional file 1: Combined report from personal genomics providers. (DOCX 275 kb) Additional file 2: Genome Trax table with homozygous deleterious SNPs and their associated disease. (XLSX 41 kb) Additional file 3: Table summary of ingenuity analysis results. (XLS 96 kb) Additional file 4: Consent form signed by the living participants of this study. (PDF 544 kb)

## References

[1] Hood L, Balling R, Auffray C (2012). Revolutionizing medicine in the 21st century through systems approaches. Biotechnol J.

[2] Hood L (2013). Systems biology and p4 medicine: past, present, and future. Rambam Maimonides Med J.

[3] Bolouri H (2008). Computational challenges of personal genomics. Curr Genomics.

[4] Personal Genome Project. [http://www.personalgenomes.org/]

[5] Glusman G, Cariaso M, Jimenez R, Swan D, Greshake B, Bhak J, Logan DW, Corpas M (2012). Low budget analysis of Direct-To-Consumer genomic testing familial data. F1000Res.

[6] Roach JC, Glusman G, Smit AFA, Huff CD, Hubley R, Shannon PT, Rowen L, Pant KP, Goodman N, Bamshad M, Shendure J, Drmanac R, Jorde LB, Hood L, Galas DJ (2010). Analysis of genetic inheritance in a family quartet by whole-genome sequencing. Science.

[7] Cariaso M, Lennon G (2012). SNPedia: a wiki supporting personal genome annotation, interpretation and analysis. Nucleic Acids Res.

[8] Roach JC, Glusman G, Hubley R, Montsaroff SZ, Holloway AK, Mauldin DE, Srivastava D, Garg V, Pollard KS, Galas DJ, Hood L, Smit AFA (2011). Chromosomal haplotypes by genetic phasing of human families. Am J Hum Genet.

[9] Coletta A, Molter C, Duqué R, Steenhoff D, Taminau J, de Schaetzen V, Meganck S, Lazar C, Venet D, Detours V, Nowé A, Bersini H, Weiss Solís DY (2012). InSilico DB genomic datasets hub: an efficient starting point for analyzing genome-wide studies in GenePattern, Integrative Genomics Viewer, and R/Bioconductor. Genome Biol.

[10] Greshake B, Bayer PE, Rausch H, Reda J (2014). openSNP--a crowdsourced web resource for personal genomics. PLoS One.

[11] Corpas M (2013). Crowdsourcing the corpasome. Source Code Biol Med.

[12] Manuel Corpas' Blog. [http://manuelcorpas.com]

[13] NCBI Resource Coordinators (2014). Database resources of the National Center for Biotechnology Information. Nucleic Acids Res.

[14] Han J, Kraft P, Nan H, Guo Q, Chen C, Qureshi A, Hankinson SE, Hu FB, Duffy DL, Zhao ZZ, Martin NG, Montgomery GW, Hayward NK, Thomas G, Hoover RN, Chanock S, Hunter DJ (2008). A genome-wide association study identifies novel alleles associated with hair color and skin pigmentation. PLoS Genet.

[15] Rees JL (2003). Genetics of hair and skin color. Annu Rev Genet.

[16] Candille SI, Absher DM, Beleza S, Bauchet M, McEvoy B, Garrison NA, Li JZ, Myers RM, Barsh GS, Tang H, Shriver MD (2012). Genome-wide association studies of quantitatively measured skin, hair, and eye pigmentation in four European populations. PLoS One.

[17] Branicki W, Liu F, van Duijn K, Draus-Barini J, Pośpiech E, Walsh S, Kupiec T, Wojas-Pelc A, Kayser M (2011). Model-based prediction of human hair color using DNA variants. Hum Genet.

[18] MacArthur DG, Balasubramanian S, Frankish A, Huang N, Morris J, Walter K, Jostins L, Habegger L, Pickrell JK, Montgomery SB, Albers CA, Zhang ZD, Conrad DF, Lunter G, Zheng H, Ayub Q, DePristo MA, Banks E, Hu M, Handsaker RE, Rosenfeld JA, Fromer M, Jin M, Mu XJ, Khurana E, Ye K, Kay M, Saunders GI, Suner M-M, Hunt T (2012). A systematic survey of loss-of-function variants in human protein-coding genes. Science.

[19] de Rijke B, van Horssen-Zoetbrood A, Beekman JM, Otterud B, Maas F, Woestenenk R, Kester M, Leppert M, Schattenberg AV, de Witte T, van de Wiel-van Kemenade E, Dolstra H (2005). A frameshift polymorphism in P2X5 elicits an allogeneic cytotoxic T lymphocyte response associated with remission of chronic myeloid leukemia. J Clin Invest.

[20] Landrum MJ, Lee JM, Riley GR, Jang W, Rubinstein WS, Church DM, Maglott DR (2014). ClinVar: public archive of relationships among sequence variation and human phenotype. Nucleic Acids Res.

[21] Liang J, Fu M, Ciociola E, Chandalia M, Abate N (2007). Role of ENPP1 on adipocyte maturation. PLoS One.

[22] Johnson K, Vaingankar S, Chen Y, Moffa A, Goldring MB, Sano K, Jin-Hua P, Sali A, Goding J, Terkeltaub R (1999). Differential mechanisms of inorganic pyrophosphate production by plasma cell membrane glycoprotein-1 and B10 in chondrocytes. Arthritis Rheum.

[23] O'Hayre M, Vázquez-Prado J, Kufareva I, Stawiski EW, Handel TM, Seshagiri S, Gutkind JS (2013). The emerging mutational landscape of G proteins and G-protein-coupled receptors in cancer. Nat Rev Cancer.

[24] Burmester JK, Suarez BK, Lin JH, Jin CH, Miller RD, Zhang K-Q, Salzman SA, Reding DJ, Catalona WJ (2004). Analysis of candidate genes for prostate cancer. Hum Hered.

[25] Kohlhase S, Bogdanova NV, Schürmann P, Bermisheva M, Khusnutdinova E, Antonenkova N, Park-Simon T-W, Hillemanns P, Meyer A, Christiansen H, Schindler D, Dörk T (2014). Mutation analysis of the ERCC4/FANCQ gene in hereditary breast cancer. PLoS One.

[26] Khan SR, Canales BK (2009). Genetic basis of renal cellular dysfunction and the formation of kidney stones. Urol Res.

[27] Garat A, Cauffiez C, Renault N, Lo-Guidice JM, Allorge D, Chevalier D, Houdret N, Chavatte P, Loriot MA, Gala JL, Broly F (2008). Characterisation of novel defective thiopurine S-methyltransferase allelic variants. Biochem Pharmacol.

[28] Relling MV, Gardner EE, Sandborn WJ, Schmiegelow K, Pui C-H, Yee SW, Stein CM, Carrillo M, Evans WE, Klein TE, Clinical Pharmacogenetics Implementation Consortium (2011). Clinical Pharmacogenetics Implementation Consortium guidelines for thiopurine methyltransferase genotype and thiopurine dosing. Clin Pharmacol Ther.

[29] Ujiie S, Sasaki T, Mizugaki M, Ishikawa M, Hiratsuka M (2008). Functional characterization of 23 allelic variants of thiopurine S-methyltransferase gene (TPMT*2 - *24). Pharmacogenet Genomics.

[30] Valdivia-Granda WA (2010). Bioinformatics for biodefense: challenges and opportunities. Biosecur Bioterror.

[31] Valdivia-Granda WA. Biosurveillance enterprise for operational awareness, a genomic-based approach for tracking pathogen virulence. Virulence. 2013;4. 10.4161/viru.26893 PMC3925708 24152965

[32] Elements of informed consent described in Federal Regulations. [http://www.fda.gov/RegulatoryInformation/Guidances/ucm126431.htm]

[33] Global Alliance for Genomics and Health. [http://genomicsandhealth.org/]

[34] Imai K, Kricka LJ, Fortina P (2011). Concordance study of 3 direct-to-consumer genetic-testing services. Clin Chem.

[35] Kalf RRJ, Mihaescu R, Kundu S, de Knijff P, Green RC, Janssens ACJW (2014). Variations in predicted risks in personal genome testing for common complex diseases. Genet Med.

[36] Ng PC, Murray SS, Levy S, Venter JC (2009). An agenda for personalized medicine. Nature.

[37] O'Rawe J, Jiang T, Sun G, Wu Y, Wang W, Hu J, Bodily P, Tian L, Hakonarson H, Johnson WE, Wei Z, Wang K, Lyon GJ (2013). Low concordance of multiple variant-calling pipelines: practical implications for exome and genome sequencing. Genome Med.

[38] Miquel S, Martín R, Rossi O, Bermúdez-Humarán LG, Chatel JM, Sokol H, Thomas M, Wells JM, Langella P (2013). Faecalibacterium prausnitzii and human intestinal health. Curr Opin Microbiol.

[39] Genomes Unzipped. [http://www.genomesunzipped.org/]

[40] Keeping it in the family. [http://news.sciencemag.org/2012/08/keeping-it-family]

[41] Father. [https://insilicodb.com/app/publicutilities/getfile?seriesName=ISDB13134&platformName=GPL9115&file=source/ISDB13134-measurements/ISDBM376876/exome_germinal/v1.0/bam_raw/ISDBM376876.sorted.bam]

[42] Mother. [https://insilicodb.com/app/publicutilities/getfile?seriesName=ISDB13134&platformName=GPL9115&file=source/ISDB13134-measurements/ISDBM376878/exome_germinal/v1.0/bam_raw/ISDBM376878.sorted.bam]

[43] Daughter. [https://insilicodb.com/app/publicutilities/getfile?seriesName=ISDB13134&platformName=GPL9115&file=source/ISDB13134-measurements/ISDBM376877/exome_germinal/v1.0/bam_raw/ISDBM376877.sorted.bam]

[44] Son. [https://insilicodb.com/app/publicutilities/getfile?seriesName=ISDB13134&platformName=GPL9115&file=source/ISDB13134-measurements/ISDBM376875/exome_germinal/v1.0/bam_raw/ISDBM376875.sorted.bam]

[45] Li H, Durbin R (2009). Fast and accurate short read alignment with Burrows-Wheeler transform. Bioinformatics.

[46] Picard. [http://broadinstitute.github.io/picard/]

[47] DePristo MA, Banks E, Poplin R, Garimella KV, Maguire JR, Hartl C, Philippakis AA, del Angel G, Rivas MA, Hanna M, McKenna A, Fennell TJ, Kernytsky AM, Sivachenko AY, Cibulskis K, Gabriel SB, Altshuler D, Daly MJ (2011). A framework for variation discovery and genotyping using next-generation DNA sequencing data. Nat Genet.

[48] Stenson PD, Mort M, Ball EV, Shaw K, Phillips AD, Cooper DN. The Human Gene Mutation Database: building a comprehensive mutation repository for clinical and molecular genetics, diagnostic testing and personalized genomic medicine. Hum Genet. 2013. 10.1007/s00439-013-1358-4 PMC3898141 24077912

[49] Welter D, MacArthur J, Morales J, Burdett T, Hall P, Junkins H, Klemm A, Flicek P, Manolio T, Hindorff L, Parkinson H (2014). The NHGRI GWAS Catalog, a curated resource of SNP-trait associations. Nucleic Acids Res.

[50] Abecasis GR, Altshuler D, Auton A, Brooks LD, Durbin RM, Gibbs RA, Hurles ME, McVean GA, 1000 Genomes Project Consortium (2010). A map of human genome variation from population-scale sequencing. Nature.

[51] Rath A, Olry A, Dhombres F, Brandt MM, Urbero B, Ayme S (2012). Representation of rare diseases in health information systems: the Orphanet approach to serve a wide range of end users. Hum Mutat.

[52] Kamphans T, Krawitz PM (2012). GeneTalk: an expert exchange platform for assessing rare sequence variants in personal genomes. Bioinformatics.

[53] Kumar P, Henikoff S, Ng PC (2009). Predicting the effects of coding non-synonymous variants on protein function using the SIFT algorithm. Nat Protoc.

[54] Adzhubei I, Jordan DM, Sunyaev SR (2013). Predicting functional effect of human missense mutations using PolyPhen-2. Curr Protoc Hum Genet.

