# Supplementary material for: Crowdsourced direct-to-consumer genomic analysis of a family quartet
Source: BMC Genomics. 2015 Nov 7;16:910. doi: 10.1186/s12864-015-1973-7 (PMC4636840; doi:10.1186/s12864-015-1973-7)
Supplement: Additional file 1: — Combined report from personal genomics providers. (DOCX 275 kb) [file 12864_2015_1973_MOESM1_ESM.docx]

Index

BIOBASE Report 2

Ingenuity Variant Analysis 5

Diploid Report 6

GeneTalk Report 22

BIOBASE Report

**Output file format explanations for Supplementary material spreadsheet Suppl_BIOBASEAnalysis.xlsx**

HGMD Variant Type: This is the primary focus for finding interesting variants, and most interesting are the disease-causing variants. Predisposing variants tend not to have complete penetrance: one can easily harbour some, without having or ever getting the disease. It means that there is some evidence from a study.

*DM* means the variant is flagged as having been reported to cause or contribute to disease. ‘*DM?’* means the same, but there are doubts about the report, due to other reports, the variant being very common, or insufficient supporting evidence in the paper. *FTV* are frameshift/truncating variants, *DFP* polymorphism associated with disease and functionally characterised, *DP* polymorphism associated with disease, *DF* polymorphisms functionally characterised.

HGMD Match exactness: *bases* means that the variant matched the known disease causing variant, with the very same base change. Sometimes the gene and thus the HGMD variant are reported on the negative strand, so the nucleotides listed in HGMD may appear to be the complementary bases to the ones in the VCF file, which reports always on the positive strand. *coords* means the variant just overlaps a known HGMD mutation, but does not show the same single nucleotide base change. We only check exact nucleotides for SNVs, any insertions/deletions are by default checked only by coords. Base matching variants are more interesting than ones just overlapping, but one really has to look at each case.

HGMD Disease: lists the disease or phenotype associated with the variant.

As HGMD does not record if a variant is causing disease in a dominant, recessive or other manner, as additional context we provide:

- Zygosity: says if the subject had one or two different alleles of the variant in the sample data.
- Inheritance: this is not specific to the variant, but is a statement about how a disease associated to the gene of the variant may be inherited, the data are from OrphaNet.
- Allele Frequency: this is from the CEU pilot of the 1000 Genomes project, we also added EVS counts for comparison. If these counts are high, it would be an indication that the variant is probably not harmful.

**Analysis commentary**

The major variants under these criteria are

1. CM060322 ALys amyloidosis, systemic in LYZ, which Son inherits also homozygously from parents who are heterozygotes, where the allele is relatively rare in the general population. It also is nonsynomyous coding, and predicted deleterious by MutPred (see below).


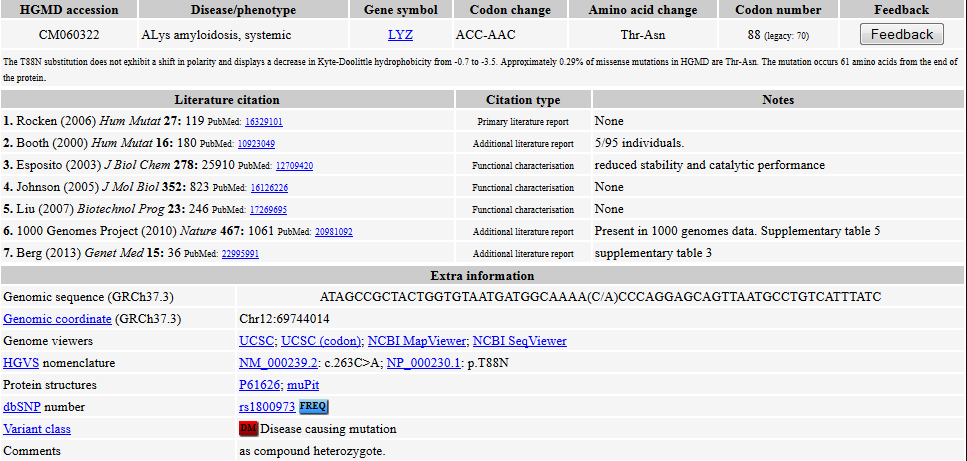


1. CD054974, Allogenic cytotoxic T lymphocyte response in P2RX5 is a frameshift deletion, again with parents being heterozygote, Son homozygous (see below).


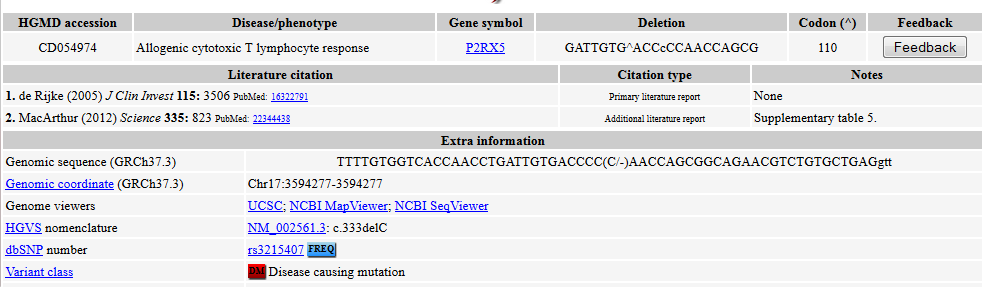


1. CS042144, Altered splicing, in CFTR, the cystic fibrosis gene. Son has a coordinate match for being homozygous for this. The variant appears because Mother has the pathogenic allele in heterozygous fashion. In any case, this is a disease-associated polymorphism (see below).


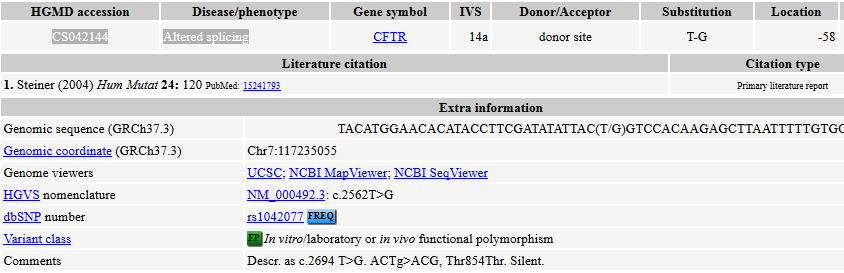


1. CM057373, Preeclampsia in STOX1. Son is homozygous, with an exact base match, for this variant. However, Preeclampsia is a condition only relevant in pregnancy.

These are the only DMs, for which Son is homozygous. There also is one variant, for which Son is heterozygous, but the gene of which has been described to pass on the disease in a dominant manner:

1. CD097476, Long QT syndrome in KCNH2. However, this is just a coordinate, match, the pathogenic mutation is a frameshift, while Son’s is merely a SNV, and a synonymous one at that, which is quite common in the population, in fact is the more common allele than the one in the reference.


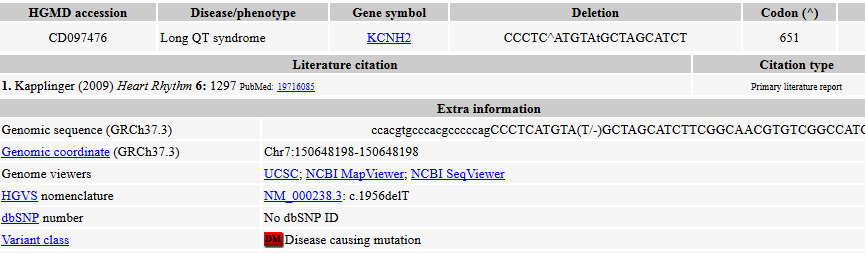


Son is a carrier of a variant in TCOF1, which is associated with Treacher-Collins syndrome, some of which variants are inherited in a dominant manner. However, Son’s allele again is relatively common, and also only matches by coordinates, so this is probably not relevant either. For predicted genotype-phenotype associations for the other members of the Corpas family please refer to supplementary Table 1 (Suppl_BIOBASEAnalysis.xlsx).

Ingenuity Variant Analysis

A series of filters used by Ingenuity variant analysis to construct a dominant inheritance model is given in Supplementary Figure S1. Variant summary and overview of the predictions are given in different spreadsheets of MS Excel file (Suppl_IngenuityVariantAnalysis.xls). Annotated variants of the dataset at *Genetic analysis* filter for dominant inheritance model (57 variants associated with 60 genes) are given in Table S2. Gene models that have one or more variants and distinct disease phenotypes associated with the variants are given in Tables S3 and S4, respectively. An enrichment analysis p-value performed based on the number of variants of each category is provided. The p-value refers to the significance of category. The pathways, gene groups or complexes and, distinct molecular and cellular processes of the variants are also given (see spreadsheet).


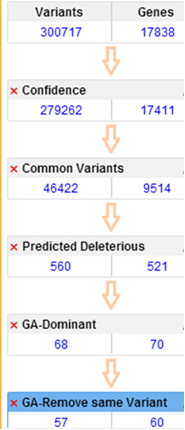


**🡨 Second Genetic Analysis (GA) filter**

**Excluded ‘homozygous’ and ‘heterozygous’ genotypes in controls and, with genotype occurrence in at least 2 of the 2 control samples at variant level.**

**🡨 Genetic analysis (GA) filter for ‘Dominant’ variants**

**Restricted to ‘transmitted’ variants and ‘heterozygous’ genotype in cases and,**

**with genotype occurrence in 2 of the 2 case samples at variant level;**

**Excluded genotypes except ‘heterozygous’ in controls and,**

**with genotype occurrence in at least 1 of the 2 control samples at variant level**

**🡨 Retained variants that were experimentally observed to be associated with a phenotype: pathogenic and likely pathogenic or associated with gain of function of a gene: established in the literature or associated with loss of function of a gene: Frameshift, in-frame indel, or start/stop codon change and missense and not predicted tolerated by SIFT or PolyPhen-2**

**🡨 Excluded variants with an allele frequency of at least 3% observed in**

**the 1000 Genomes Project, public Complete Genomics genomes and**

**Exome Sequencing Project of the National Heart Lung and Blood Institute**

**🡨 Filtered variants of call quality < 20 in samples, outside top 5% most exonically variable 100base windows in healthy public genomes, and outside top 1% most exonically variable genes in 1000 genomes**

**Supplementary Figure S1**: Filter cascade used for variant analysis on four samples Sister, Son, Father and Mother. The series of filters used are shown: ‘Confidence’, ‘Common Variants’, ‘Predicted Deleterious’ and ‘Genetic Analysis’ for dominant inheritance model. Under each filter the number to the left indicates the number of variants that remain in the dataset after applying the respective filter and all filters above it. The number on the right indicates the number of distinct genes within which those variants are observed.

Diploid Report

**SNPs of unconfirmed significance**

SNPs for which a preliminary association with HIV-resistance and thiopurine toxicity has been reported (but overall currently of unknown clinical relevance):

**HIV resistance**

All four family members might be more resistant to infection with the most common strain of HIV (HIV-1). This is indicated by the heterozygous CT or homozygous TT calls for Mother, Father, and Son and Daughter, respectively, for the rs3775291 polymorphism within the *TLR3* gene. TLR3 contributes in the activation of antiviral immune responses. The L412F polymorphism was recently shown to have a protective effect against HIV infection, in a dominant fashion. However, given the limited amount of data currently available, the impact of the rs3775291 polymorphism on HIV resistance is still preliminary.

1. Sironi M, Biasin M, Cagliani R, Forni D, De Luca M, Saulle I, Lo Caputo S, Mazzotta F, Macías J, Pineda JA, Caruz A, Clerici M. (2012). A common polymorphism in TLR3 confers natural resistance to HIV-1 infection. J Immunol., 188(2):818-23. doi: 10.4049/jimmunol.1102179.
2. Huik K, Avi R, Pauskar M, Kallas E, Jõgeda EL, Karki T, Marsh K, Des Jarlais D, Uusküla A, Lutsar I (2013). Association between TLR3 rs3775291 and resistance to HIV among highly exposed Caucasian intravenous drug users. Infection, Genetics and Evolution, 20: 78–82.

**Thiopurine toxicity**

Father and Daughter both carry one normal and one variant copy of the *TPMT* gene, denoted as *TPMT*9* (rs151149760). TPMT, or thiopurine methyltransferase is an enzyme that inactivates thiopurine drugs, which are used to treat certain cancers of the bone marrow, various auto-immune diseases and transplant patients. Individuals with reduced activity of the TPMT enzyme are more likely to suffer from toxic effects following treatment with conventional doses of these drugs. The exact functional status and pharmacogenomic implications of the extremely rare *TPMT**9 allele are still preliminary. Although TPMT*9 has been suggested to have reduced enzyme activity and possible implications for thiopurine toxicity, the allele has both been detected in patients with an intermediate TPMT phenotype as well as in a patient with normal TPMT activity.

1. Garat, A., Cauffiez, C., Renault, N., Lo-Guidice, J. M., Allorge, D., Chevalier, D., et al. (2008). Characterisation of novel defective thiopurine S-methyltransferase allelic variants. Biochemical pharmacology, 76(3), 404–415. doi:10.1016/j.bcp.2008.05.009
2. Relling, M. V., Gardner, E. E., Sandborn, W. J., Schmiegelow, K., Pui, C.-H., Yee, S. W., et al. (2011). Clinical Pharmacogenetics Implementation Consortium Guidelines for Thiopurine Methyltransferase Genotype and Thiopurine Dosing. Clinical Pharmacology & Therapeutics, 89(3), 387–391. doi:10.1038/clpt.2010.320
3. Ujiie, S., Sasaki, T., Mizugaki, M., Ishikawa, M., & Hiratsuka, M. (2008). Functional characterization of 23 allelic variants of thiopurine S-methyltransferase gene (TPMT*2 – *24). Pharmacogenetics and Genomics, 18(10), 887–893. doi:10.1097/FPC.0b013e3283097328

**PRSS1-related hereditary pancreatitis**

Although we cannot draw conclusions from the variants identified in this analysis, we were intrigued when finding several variants within the *PRSS1* gene for all four family members.

The standard Diploid analysis identified rs111033566 within *PRRS1* as heterozygous for all four family members, a SNP which has been repeatedly reported as being pathogenic for *PRSS1*-related Hereditary Pancreatitis. As mentioned in the previous report, we assumed incomplete penetrance contributed to the fact that none of the family members were affected. However, the results from the extended analysis of the exome data now raise some doubt about the reliability of the heterozygous call for rs111033566, as well as the other calls in *PRSS1*.

In addition to rs111033566, all four family members have heterozygous calls for some other, positions within the *PRSS1* gene (currently of unknown significance). Although the coverage depth is high for all calls (as it was for rs111033566), there is an imbalance in allelic depth. For heterozygous calls, we would expect an equal amount of sequences containing the reference or alternative allele (giving an 1:1 ratio). However, for all heterozygous calls within *PRSS1*, this ratio deviates from the expected 1:1 ratio.

| **PRSS1 SNP/position** | **Family member** | **Genotype** | **Allelic depth (Ref/Alt)** |
| --- | --- | --- | --- |
| **rs111033566** | Father | Heterozygous GA | 56/37 |
|  | Mother | Heterozygous GA | 58/32 |
|  | Daughter | Heterozygous GA | 64/31 |
|  | Son | Heterozygous GA | 76/60 |
| **142459679** | Father | Heterozygous GA | 63/21 |
|  | Mother | Heterozygous GA | 55/31 |
|  | Daughter | Heterozygous GA | 66/30 |
|  | Son | Heterozygous GA | 140/28 |
| **142460339** | Father | Heterozygous GA | 147/15 |
|  | Mother | Heterozygous GA | 132/27 |
|  | Daughter | Heterozygous GA | 135/42 |
|  | Son | Homozygous Ref. | - |
| **142460369** | Father | Heterozygous GA | 69/49 |
|  | Mother | Heterozygous GA | 71/36 |
|  | Daughter | Heterozygous GA | 69/49 |
|  | Son | Heterozygous GA | 105/52 |
| **142460394** | Father | Heterozygous TC | 65/25 |
|  | Mother | Heterozygous TC | 68/15 |
|  | Daughter | Heterozygous TC | 77/17 |
|  | Son | Heterozygous TC | 103/22 |

We therefore had another look at the data. In particular, we looked at the accuracy of the calls predicted by the Variant Quality Score Recalibration, which gives a single estimate for the accuracy of each call by comparing the VQSLOD scores of each variant with a calculated threshold value (for detailed info on this parameter, see http://gatkforums.broadinstitute.org/discussion/39/variant-quality-score-recalibration-vqsr). Variants that are above the threshold are considered of good accuracy (‘PASS’ in filter column), variants for which VQSLOD falls below the threshold, are considered false positives.

In the vcf-files, there is only information about this filtering step for one of the samples. However, all variants within *PRSS1* (also rs111033566 that came out of the standard analysis) seem to be false positive calls, based on this threshold. Although it is not clear why the *PRSS1* calls all turn out to be false positive.

In conclusion, the positive result for Hereditary pancreatitis is probably due to a false positive call for rs111033566.

Comments and details for the other rare variants are given below. Variants that seem to be accurately called are indicated in orange, however, the variants remain of unknown clinical significance.

**SON**

Red (Polyphen and Sift scores above the threshold)

1. DYSF (only found in Son): CT heterozygous: potentially related to a carrier status of autosomal recessive limb girdle muscular dystrophy 2B or Miyoshi myopathy, Sequencing depth 17 ok, imbalance in allele depth (13:4), PASS, GQ ok (99), Phred likelihood (102, 0, 409); **based on the imbalance in allele depth, the alternative base call might be inaccurate**.
2. SLC25A20 (only found in Son): CA heterozygous: potentially related to a carrier status of carnitine-acylcarnitine translocase deficiency, a metabolic disorder. allele depth (1:2), low sequencing depth (3), low GQ (27), PASS, PL (66, 0, 27), **based on GQ and sequencing depth probably an inaccurate call**.
3. HNF1A (idem Mother): GA heterozygous: potentially pathogenic for MODY3 (dominant), low sequencing depth (4), low allele depth (1:3), low GQ, PASS, PL (107, 0, 17), **based on GQ and low sequencing depth probably an inaccurate call**.
4. LAMA3 (idem Daughter and Mother): CA heterozygous: potentially related to a carrier status of epidermolysis bullosa, a skin disorder (ranges from mild to severe), sequencing depth (23) seems ok, allele depth (13,10) ok, GQ 99 ok, PASS, PL (294, 0, 425), **seems to be an accurate call, however the variant itself remains of unknown clinical significance**.
5. RHPN2 (idem Daughter and Father): TC heterozygous: this gene has not yet been shown to be associated with a genetic disease or trait, its protein product plays a role in the organisation of the actin cytoskeleton. sequencing depth (69) ok, allele depth (43, 26) imbalance?, GQ 99 ok, PL (440, 0, 778), VQSRTrancheSNP99.90to100.00 (though not clear whether this applies to the sample of the Son), **based on VQSR and allele imbalance probably a false positive call**.

Orange (Sift or Polyphen score above the threshold):

1. HSPD1 (idem Daughter and Mother): CT heterozygous: potentially related to a carrier status of autosomal recessive spastic paraplegia 13, polyphen 0,85, sequencing depth (41) ok, allele depth (32:9) imbalance?, GQ 99 ok, PL (174, 0, 1112), VQSRTrancheSNP99.00to99.90, **based on VQSR and allele imbalance probably a false positive call**.
2. ARX (idem Mother?; in vcf Mother genotype ./. is indicated): TT homozygous alt: potentially pathogenic for X-linked mental retardation and epilepsy. Sift 0,03, low sequencing depth (2), allele depth (0:2), low GQ (6), PL (78, 6, 0), PASS (! in vcf genotype: 1/1, this gene is located on the X-chormosome, call needs to be haploid for the son, thus carries the alt allele T according to the vcf), **based on low sequencing depth and low GQ probably an inaccurate call**.

Grey:

did not pass the Sift and/or Polyphen thresholds + no reliable homozygous calls

White (no Sift and/or Polyphen scores available):

1. CLCNKA (only found in Son): TC heterozygous: potentially related to a carrier status of Bartter syndrome with sensorineural deafness, a kidney disorder combined with sensorineural deafness, sequencing depth (34) ok, allele depth (27:7) imbalance, low GQ (71), PL (71, 0, 637), VQSRTrancheSNP99.90to100.00, **based on VQSR and allele imbalance probably a false positive call**.
2. PYHIN1 (idem Daughter and Father): CT heterozygous: this gene has not yet clearly been correlated to a certain disease or trait, it functions as a tumor suppressor, in that respect down regulation of this gene is associated with breast cancer, sequencing depth: 11 rather low, allele depth (4:7) imbalance, GQ 99 ok, PL (235, 0, 123), PASS, **based on imbalance in allele depth probably an inaccurate call, though GQ and VQSR seem ok**.
3. FCGR2B (idem Daughter & Father, both very low GQ): TG heterozygous: this gene has not yet clearly been correlated to a certain disease or trait, it is involved in phagocytosis of immune complexed and antigen production regulation, variations might increase susceptibility to systemic lupus erythematosus. Sequencing depth (19) ok, allele depth (14, 5) imbalance, GQ 12 low, PL (12, 0, 140), VQSRTrancheSNP99.90to100.00, **Based on VQSR, allele imbalance and low GQ probably a false positive call**.
4. PKP1 (idem Daughter & Mother, both very low GQ): GT heterozygous: potentially related to a carrier status of ectodermal dysplasia - skin fragility syndrome, a skin disorder. sequencing depth (2) low, allele depth (1:1), low GQ (32), PL (32, 0, 34), PASS, **Based on the low sequencing depth and low GQ probably an inaccurate call**.
5. ALMS1 (idem Father): AG heterozygous: potentially related to a carrier status of Alström syndrome, a multi systemic disorder, characterised by childhood obesity, blindness, hearing loss and other symptoms. polyphen (0,985), sequencing depth (18) +/- ok, allele depth (11, 7) ok, GQ 99 ok, PL (222,0,367), PASS, **Seems to be an accurate call, however, the variant itself remains of unknown clinical significance**.
6. ZAP70 (only found in Son): CA heterozygous: potentially related to a carrier status of ZAP70-related severe combined immunodeficiency, an immune disorder. Sequencing depth (24) ok, allele depth (20, 4) imbalance, GQ (81) rather low, PL (81, 0, 677), polyphen 0,988, VQSRTrancheSNP99.00to99.90, **Based on VQSR and allele imbalance probably a false positive call**.
7. HS6ST1 (idem Daughter & Mother, but both low GQ): GA heterozygous: This gene has not yet been associated with a certain disease or trait, it functions as an enzyme involved in generating heparin sulcate fine structures. Sequencing depth (60) ok, allele depth (49,11) imbalance, low GQ (5), PL (5, 0, 558), VQSRTrancheSNP99.90to100.00, **Based on allele imbalance, VQSR and low GQ probably a false positive call**.
8. HS6ST1 (idem Mother & Father, but both low GQ): GT heterozygous: see previous one, sequencing depth (28) ok, allele depth (12,16) ok, GQ 99 ok, PL (207, 0, 102), VQSRTrancheSNP99.90to100.00, **Based on VQSR probably a false positive call**.
9. … (low GQ, VQSR, low depth, allele imbalance, no genotype (./.) …)
10. SMARCAL1 (idem Daughter and Father): CT heterozygous, potentially related to a carrier status of Schimke immuno-osseous dysplasia, characterised by short stature, kidney disease and a weakened immune system. Sequencing depth (54) ok, allele depth (29, 25) ok, GQ (99) ok, PL (785, 0, 892), PASS, **seems to be an accurate call, however the variant itself remains of unknown clinical significance + unknown Sift and polyphen scores.**
11. MANBA (idem Daughter & Father, but in both samples no genotype call): TT homozygous: potentially pathogenic for beta-mannosidosis, a lysososmal storage disease with neurological involvement. sequencing depth (2) low, allele depth (0,2) low, (! genotype in vcf: **1/1???**), GQ (6) low, PL (79,6,0), PASS, **Based on low sequencing depth and GQ probably an inaccurate call**.
12. LAMA2 (idem Daughter & Mother): CT heterozygous: potentially related to a carrier status of congenital merosin-deficient muscular dystrophy. Sequencing depth: 49 ok, allele depth (28;21) ok, GQ: 99, PL (643, 0, 907), PASS, **seems to be an accurate call, however the variant itself remains of unknown clinical significance + unknown Sift and polyphen scores.**
13. SYNE1 (idem Father): GCTTG heterozygous: potentially related to a carrier status of autosomal recessive cerebellar ataxia type 1. Sequencing depth (64) ok, allele depth (33,30), GQ 99 ok, PL (1451, 0, 1264), PASS, **seems to be an accurate call, however the variant itself remains of unknown clinical significance + unknown Sift and polyphen scores.**
14. CNTNAP2 (idem Daughter & Mother, low GQ in sample daughter): TC heterozygous: this gene has not yet been correlated to a certain disease or trait, it plays a role as cell adhesion molecules and receptors in neurons, it has been implicated in some neurodevelopmental disorders such as Gilles de la Tourette syndrome, schizophrenia, epilepsy, autism, ADHD, and mental retardation. Sequencing depth (41) ok, allele depth (31,10) imbalance, GQ 99, pL (242,0,910), PASS, **seems to be an accurate call, however the variant itself remains of unknown clinical significance + unknown Sift and polyphen scores.**
15. DPYS (idem Daughter & Mother): AG heterozygous: potentially related to a carrier status of dihydropyriminidase deficiency, ranges from asymptomatic to neurological problems. Sequencing depth (33) ok, allele depth (20,13) imbalance?, GQ 99 ok, PL (392,0,651), PASS, **seems to be an accurate call, however the variant itself remains of unknown clinical significance + unknown Sift and polyphen scores.**
16. HOGA1 (idem Daughter & Father) : CT heterozygous: potentially related to a carrier status of primary hyperoxaluria type 3, which can cause kidney damage. sequencing depth (61) ok, allele depth (24,37) imbalance?, GQ99 ok, PL (973,0,788), PASS, **seems to be an accurate call, however the variant itself remains of unknown clinical significance + unknown Sift and polyphen scores.**
17. SLCO1B3 (idem Father): TA heterozygous: this gene has been associated with Rotor type hyperbilirubinemia, it functions as a transporter in the liver and plays a role in bile acid and bilirubin transport. Sequencing depth (59) ok, allele depth (44,15) imbalance, GQ 99 ok, PL (413,0,1479), PASS, Polyphen score 1, **seems to be an accurate call, however the variant itself remains of unknown clinical significance + allele imbalance?.**
18. EIF2B2 (idem Daughter & Father): AG heterozygous: potentially related to a carrier status of leukoencephalopathy with vanishing white matter, a progressive neurological disorder. Sequencing depth (86) ok, allele depth (55,31) imbalance, GQ 99 ok, PL (940, 0, 1812), Polyphen score 0,948, **seems to be an accurate call, however the variant itself remains of unknown clinical significance + allele imbalance.**
19. IFT140 (idem Daughter & Mother): CT heterozygous: this gene has not yet been correlated to a certain disease or trait, plays a role in cilia function (potential role in kidney function). Sequencing depth (50) ok, allele depth (30,20) ok, GQ 99 ok, PL (618,0,869), PASS, **seems to be an accurate call, however the variant itself remains of unknown clinical significance + unknown Sift and polyphen scores.**
20. DNM2 (idem Father): CT heterozygous: potentially pathogenic for centronuclear myopathy, that affects skeletal muscles, or Charcot-Marie Tooth disease, a neurological disorder with progressive muscle weakness. sequencing depth (27) ok, allele depth (13,14) ok, GQ 99 ok, PL (371,0,308), PASS, **seems to be an accurate call, however the variant itself remains of unknown clinical significance + unknown Sift and polyphen scores.**
21. COL6A2 (idem Daughter & Father, daughter low GQ and depth): AAC heterozygous: potentially pathogenic for Bethlem myopathy or related to a carrier status of Ullrich congenital muscular dystrophy, sequencing depth: 47 ok, allele depth (25,21) ok, GQ 99 ok, PL (823,0,634), PASS, **seems to be an accurate call, however the variant itself remains of unknown clinical significance + unknown Sift and polyphen scores.**
22. XPNPEP3 (idem Daughter & Father): TTA heterozygous: potentially related to a carrier status of nephronophthisis-like nephropathy, a kidney disease. Sequencing depth (78) ok, allele depth (47, 30), imbalance?, GQ 99 ok, PL 1229,0,1829, PASS, **seems to be an accurate call, however the variant itself remains of unknown clinical significance + unknown Sift and polyphen scores.**

**DAUGHTER**

Red (Polyphen and Sift scores above the threshold):

1. CACNA1S (only found in Daughter): GA heterozygous: potentially pathogenic for hypokalemic periodic paralysis and/or malignant hyperthermia susceptibility. Sequencing depth: 3 low, allele depth (1,2) low, GQ: 31, PL (69,0,31), PASS, **Based on low sequencing depth and GQ probably an inaccurate call**.
2. WDPCP (idem Mother): AT heterozygous: potentially related to a carrier status of Meckel-Gruber syndrome, and/or Bardet-Biedl syndrome, two developmental disorders. Sequencing depth 22 ok, allele depth (9,13) ok, GQ 99 ok, PL (421,0,280), PASS, **seems to be an accurate call, however the variant itself remains of unknown clinical significance**.
3. SERPINI1 (idem Father): GA heterozygous: potentially pathogenic for familial encephalopathy with neuroserpin inclusion bodies, a progressive neurodegenerative disorder. Sequencing depth 38 ok, allele depth (25,13) imbalance?, GQ 99 ok, PL (371, 0,853), VQSRTrancheSNP99.00to99.90, **Based on VQSR and allele depth probably a false positive call**.
4. CCHCR1 (idem Father): GC heterozygous: this gene has not yet been associated with a certain disease or trait (except preliminary nevirapine hypersensitivity and psoriasis susceptibility), it encodes coiled-coil alpha helical rod protein 1. Sequencing depth: 26 ok, allele depth (12,14) ok, GQ 99 ok, PL (375,0,363), PASS, **seems to be an accurate call, however the variant itself remains of unknown clinical significance**.
5. LAMA3 (idem Son & Mother): CA heterozygous: potentially related to a carrier status of epidermolysis bullosa, a skin disorder (ranges from mild to severe), sequencing depth (21) seems ok, allele depth (15,6) imbalance, GQ 99 ok, PASS, PL (162, 0, 533), **seems to be an accurate call, however the variant itself remains of unknown clinical significance**.
6. RHPN2 (idem Son & Father): TC heterozygous: this gene has not yet been shown to be associated with a genetic disease or trait, its protein product plays a role in the organisation of the actin cytoskeleton. sequencing depth (29) ok, allele depth (20, 9) imbalance, GQ 56 low, PL (56, 0, 476), VQSRTrancheSNP99.90to100.00, **based on VQSR, low GQ and allele imbalance probably a false positive call**.

Orange (Sift or Polyphen score above the threshold):

1. HSPD1 (idem Son & Mother): CT heterozygous: potentially related to a carrier status of autosomal recessive spastic paraplegia 13, polyphen 0,85, sequencing depth (50) ok, allele depth (44:6) imbalance, GQ 74 low, PL (74, 0, 1582), VQSRTrancheSNP99.00to99.90, **based on VQSR, low GQ and allele imbalance probably a false positive call**.
2. ABCC3 (idem Mother, but low GQ): AC heterozygous: This gene has not yet been associated to a certain disease or trait, functions as a transporter, potentially involved in multidrug resistance, Sequencing depth: 9 low, allele depth: 8:1, low, GQ 16 low, PL (16,0,219), PASS, **Based on low sequencing depth, GQ and allele imbalance, probably an inaccurate call**.
3. CD177 (idem Mother): GC heterozygous: this gene has not yet been associated to a certain disease or trait, encodes a cell surface glycoprotein, was first described in a case of neonatal alloimmune neutropenia. Sequencing depth (18) ok, allele depth (9:9) ok, GQ 99 ok, PL (253,0,273), PASS, **seems to be an accurate call, however the variant itself remains of unknown significance**.
4. NLRP12 (idem Mother): CG heterozygous: potentially pathogenic for familial cold auto inflammatory syndrome, which causes episodes of fever, skin rash and joint pain after exposure to cold temperatures. Sequencing depth (19) ok, allele depth (11,8) ok, GQ 99 ok, PL (225,0,363), PASS, **seems to be an accurate call, however the variant itself remains of unknown significance**.

Grey:

did not pass the Sift and/or Polyphen thresholds + no reliable homozygous calls

White (no Sift and/or Polyphen scores available): suspicious:

1. PYHIN1 (idem Son & Father): CT heterozygous: this gene has not yet clearly been correlated to a certain disease or trait, it functions as a tumor suppressor, in that respect down regulation of this gene is associated with breast cancer, sequencing depth: 28 ok, allele depth (19:9) imbalance, GQ 99 ok, PL (264, 0, 672), PASS, **based on imbalance in allele depth probably an inaccurate call, though GQ and VQSR seem ok**.
2. NEB (idem Mother): GA heterozygous: potentially related to a carrier status of nemaline myopathy, characterised by muscle weakness, scoliosis and joint deformities. Sequencing depth (53) ok, allele depth (31, 22) imbalance?, GQ 99, PL (631,0,1066), PASS, **seems to be an accurate call, however the variant itself remains of unknown clinical significance + unknown Sift and polyphen scores.**
3. SMARCAL1 (Idem Son & Father): CT heterozygous, potentially related to a carrier status of Schimke immuno-osseous dysplasia, characterised by short stature, kidney disease and a weakened immune system. Sequencing depth (42) ok, allele depth (23, 19) ok, GQ (99) ok, PL (541, 0, 782), PASS, **seems to be an accurate call, however the variant itself remains of unknown clinical significance + unknown Sift and polyphen scores.**
4. CUL7 (Idem Father): CG heterozygous, potentially related to a carrier status of 3-M syndrome type 1, a cause of dwarfism. Sequencing depth (17) ok, allele depth (8,9) ok, GQ 99 ok, PL (272, 0, 255), PASS, **seems to be an accurate call, however the variant itself remains of unknown clinical significance + unknown Sift and polyphen scores.**
5. RIMS1 (idem Father): GA heterozygous, potentially pathogenic for cone-rod dystrophy, autosomal dominant, a hereditary eye condition. Sequencing depth (29) ok, allele depth (12,17) ok, GQ 99 ok, PL (529,0,360), PASS, **seems to be an accurate call, however the variant itself remains of unknown clinical significance + unknown Sift and polyphen scores.**
6. LAMA2 (idem Son & Mother): CT heterozygous: potentially related to a carrier status of congenital merosin-deficient muscular dystrophy. Sequencing depth: 38 ok, allele depth (25;13) imbalance?, GQ: 99, PL (404, 0, 881), PASS, **seems to be an accurate call, however the variant itself remains of unknown clinical significance + unknown Sift and polyphen scores.**
7. DPYS (idem Son & Mother): AG heterozygous: potentially related to a carrier status of dihydropyriminidase deficiency, ranges from asymptomatic to neurological problems. Sequencing depth (34) ok, allele depth (21,13) imbalance?, GQ 99 ok, PL (389,0,718), PASS, **seems to be an accurate call, however the variant itself remains of unknown clinical significance + unknown Sift and polyphen scores.**
8. HOGA1 (idem Son & Father): CT heterozygous: potentially related to a carrier status of primary hyperoxaluria type 3, which can cause kidney damage. sequencing depth (19) ok, allele depth (14,5) imbalance?, GQ99 ok, PL (133,0,486), PASS, **seems to be an accurate call, however the variant itself remains of unknown clinical significance + unknown Sift and polyphen scores.**
9. WNK1 (idem Mother): TC heterozygous: potentially related to a carrier status of hereditary sensory and autonomic neuropathy type 2, impairment of pain sensations, and/or pseudohypoaldosteronism type 2. Sequencing depth (39) ok, allele depth (21,18) ok, GQ 99 ok, PL (581,0,653), PASS, **seems to be an accurate call, however the variant itself remains of unknown clinical significance + unknown Sift and polyphen scores.**
10. FGD4 (idem Mother): CT heterozygous: potentially related to a carrier status of Charcot-Marie-Tooth disease type 4H, a neurodegenerative disorder. Sequencing depth (35) ok, allele depth (20,15) ok, GQ 99 ok, PL (427, 0, 590), PASS, **seems to be an accurate call, however the variant itself remains of unknown clinical significance + unknown Sift and polyphen scores.**
11. EIF2B2 (idem Son & Father): AG heterozygous: potentially related to a carrier status of leukoencephalopathy with vanishing white matter, a progressive neurological disorder. Sequencing depth (76) ok, allele depth (42,34) imbalance?, GQ 99 ok, PL (1114, 0, 1417), Polyphen score 0,948, PASS **seems to be an accurate call, however the variant itself remains of unknown clinical significance + allele imbalance?**
12. PIF1 (idem Father): AC heterozygous: This gene has not yet been associated with a certain disease or trait, it functions as a DNA helicase that negatively regulates telomerase, a reverse transcriptase that maintains telomere length. Sequencing depth (9) rather low, allele depth (4,5) ok, GQ 99, PL (154,0,131), PASS, **seems to be an accurate call, however the variant itself remains of unknown clinical significance + unknown Sift and polyphen scores.**
13. IFT140 (idem Son & Mother): CT heterozygous: this gene has not yet been correlated to a certain disease or trait, plays a role in cilia function (potential role in kidney function). Sequencing depth (12) ok, allele depth (7,5) ok, GQ 99 ok, PL (153,0,215), PASS, **seems to be an accurate call, however the variant itself remains of unknown clinical significance + unknown Sift and polyphen scores.**
14. HNF1B (idem Father): GT heterozygous: potentially pathogenic for MODY5 and/or renal cysts. Sequencing depth: 14 ok, allele depth (8;6) ok, GQ 99 ok, PL (196,0,257), PASS, Sift 0,05, **seems to be an accurate call, however the variant itself remains of unknown clinical significance + unknown polyphen scores.**
15. XPNPEP3 (idem Son & Father): TTA heterozygous: potentially related to a carrier status of nephronophthisis-like nephropathy, a kidney disease. Sequencing depth (74) ok, allele depth (36, 38), ok, GQ 99 ok, PL 1672,0,1414, PASS, **seems to be an accurate call, however the variant itself remains of unknown clinical significance + unknown Sift and polyphen scores.**
16. **S**HROOM4 (idem Father): CT heterozygous: potentially related to a carrier status of X-linked mental retardation. Sequencing depth (57) ok, allele depth (26,31) ok, GQ 99 ok, PL (1023,0,839), PASS, **seems to be an accurate call, however the variant itself remains of unknown clinical significance + unknown Sift and polyphen scores.**

**MOTHER**

Red (Polyphen and Sift scores above the threshold):

1. WDPCP (idem daughter): AT heterozygous: potentially related to a carrier status of Meckel-Gruber syndrome, and/or Bardet-Biedl syndrome, two developmental disorders. Sequencing depth 28 ok, allele depth (18,10) ok, GQ 99 ok, PL (296,0,608), PASS, **seems to be an accurate call, however the variant itself remains of unknown clinical significance**.
2. AGGF1 (only found in Mother): GG homozygous: potentially pathogenic for Klippel-Trenaunay syndrome. Sequencing depth 4 low, allele depth (0:4), GQ 12 low, PL (145,12,0), PASS, **based on low sequencing depth and GQ probably an inaccurate call**.
3. SYNE1 (only found in Mother): GA heterozygous : potentially related to a carrier status of autosomal recessive spinocerebellar ataxia 8. Sequencing depth 11 rather low, allele depth (5,6) ok, GQ 99 ok, PL (183,0,138), PASS, **seems to be an accurate call, however the variant itself remains of unknown clinical significance**.
4. LRRC14 (only found in Mother): GA heterozygous: this gene has not yet been associated to a certain disease or trait, it encodes a leucine-rich containing protein. Sequencing depth 16 ok, allele depth (8;7) ok, GQ 99, PL (233,0,180), PASS, **seems to be an accurate call, however the variant itself remains of unknown clinical significance**.
5. HNF1A (idem Son): GA heterozygous: potentially pathogenic for MODY3 (dominant), low sequencing depth (4), low allele depth (2:2), low GQ 61, PASS, PL (66, 0, 61), **based on GQ and low sequencing depth: inaccurate call**.
6. LAMA3 (idem Son and Daughter): CA heterozygous: potentially related to a carrier status of epidermolysis bullosa, a skin disorder (ranges from mild to severe), sequencing depth (39) seems ok, allele depth (17,22) ok, GQ 99 ok, PASS, PL (672, 0, 529), **seems to be an accurate call, however the variant itself remains of unknown clinical significance**.
7. DLL3 (only found in Mother): TT homozygous: potentially pathogenic for spondylocostal dysostosis, characterised by abnormal development of the spine and ribs. Sequencing depth: 2 low, allele depth (0,2) low, GQ 6 low, PL (73,6,0), PASS, **Based on low GQ and sequencing depth probably an inaccurate call**.
8. TAZ (only found in Mother): TT homozygous: potentially pathogenic for 3-Methylglutaconic aciduria type 2, or Barth syndrome, characterized by an enlarged and weakened heart, recurrent infections, muscle weakness and delayed growth. Sequencing depth 2 low, allele depth (0,2) low, GQ 6 low, PL (70,6,0), PASS, **Based on low GQ and sequencing depth probably an inaccurate call**.

Orange (Sift or Polyphen score above the threshold):

1. HSPD1 (idem Son and Daughter): CT heterozygous: potentially related to a carrier status of autosomal recessive spastic paraplegia 13, polyphen 0,85, sequencing depth (40) ok, allele depth (33:7) imbalance, GQ 99 ok, PL (130, 0, 1114), VQSRTrancheSNP99.00to99.90, **based on VQSR and allele imbalance probably a false positive call**.
2. ABCC3 (idem Daughter): AC heterozygous: This gene has not yet been associated to a certain disease or trait, functions as a transporter, potentially involved in multidrug resistance, Sequencing depth: 8 low, allele depth: 4:4, low, GQ 75 low, PL (75,0,120), PASS, **Based on low sequencing depth and GQ, probably an inaccurate call**.
3. CD177 (idem daughter): GC heterozygous: this gene has not yet been associated to a certain disease or trait, encodes a cell surface glycoprotein, was first described in a case of neonatal alloimmune neutropenia. Sequencing depth (14) ok, allele depth (6:8) ok, GQ 99 ok, PL (240,0,187), PASS, **seems to be an accurate call, however the variant itself remains of unknown significance**.
4. NLRP12 (idem daughter): CG heterozygous: potentially pathogenic for familial cold auto inflammatory syndrome, which causes episodes of fever, skin rash and joint pain after exposure to cold temperatures. Sequencing depth (11) rather low, allele depth (8,3) imbalance?, GQ 87 low ok, PL (87,0,283), PASS, **seems to be an accurate call, however the variant itself remains of unknown significance**.

Grey:

did not pass the Sift and/or Polyphen thresholds + no reliable homozygous calls

White (no Sift and/or Polyphen scores available): suspicious:

1. NEB (idem Daughter): GA heterozygous: potentially related to a carrier status of nemaline myopathy, characterised by muscle weakness, scoliosis and joint deformities. Sequencing depth (51) ok, allele depth (25, 26) ok, GQ 99, PL (756,0,793), PASS, **seems to be an accurate call, however the variant itself remains of unknown clinical significance + unknown Sift and polyphen scores.**
2. LAMA2 (idem Son and Daughter): CT heterozygous: potentially related to a carrier status of congenital merosin-deficient muscular dystrophy. Sequencing depth: 36 ok, allele depth (22;14) imbalance?, GQ: 99, PL (442, 0, 757), PASS, **seems to be an accurate call, however the variant itself remains of unknown clinical significance + unknown Sift and polyphen scores.**
3. CNTNAP2 (idem Son (& daugher, low GQ)): TC heterozygous: this gene has not yet been correlated to a certain disease or trait, it plays a role as cell adhesion molecules and receptors in neurons, it has been implicated in some neurodevelopmental disorders such as Gilles de la Tourette syndrome, schizophrenia, epilepsy, autism, ADHD, and mental retardation. Sequencing depth (16) ok, allele depth (9,7) ok, GQ 99, pL (205,0,292, PASS, **seems to be an accurate call, however the variant itself remains of unknown clinical significance + unknown Sift and polyphen scores.**
4. DPYS (idem Son and Daughter): AG heterozygous: potentially related to a carrier status of dihydropyriminidase deficiency, ranges from asymptomatic to neurological problems. Sequencing depth (31) ok, allele depth (12,19) imbalance?, GQ 99 ok, PL (601,0,371), PASS, **seems to be an accurate call, however the variant itself remains of unknown clinical significance + unknown Sift and polyphen scores.**
5. WNK1 (idem Daughter): TC heterozygous: potentially related to a carrier status of hereditary sensory and autonomic neuropathy type 2, impairment of pain sensations, and/or pseudohypoaldosteronism type 2. Sequencing depth (29) ok, allele depth (19,10) imbalance?, GQ 99 ok, PL (246,0,557), PASS, **seems to be an accurate call, however the variant itself remains of unknown clinical significance + unknown Sift and polyphen scores.**
6. FGD4 (idem daughter): CT heterozygous: potentially related to a carrier status of Charcot-Marie-Tooth disease type 4H, a neurodegenerative disorder. Sequencing depth (44) ok, allele depth (24,20) ok, GQ 99 ok, PL (603, 0, 611), PASS, **seems to be an accurate call, however the variant itself remains of unknown clinical significance + unknown Sift and polyphen scores.**
7. **b** (only found in Mother): AT heterozygous: potentially pathogenic for familial breast and ovarian cancer. Sequencing depth 39 ok, allele depth (15,24) imbalance?; GQ 99 ok, PL (737,0,443), PASS, **seems to be an accurate call, however the variant itself remains of unknown clinical significance + unknown Sift and polyphen scores.**
8. SYNE2 (only found in Mother): GA heterozygous: this gene has not yet been associated with a certain disease or trait, the encoded protein maintains the structural integrity of the nucleus. Sequencing depth 17 ok, allele depth (11,6) imbalance?; GQ 99 ok, PL (187,0,379), PASS, **seems to be an accurate call, however the variant itself remains of unknown clinical significance + unknown Sift and polyphen scores.**
9. IFT140 (idem Son and daughter): CT heterozygous: this gene has not yet been correlated to a certain disease or trait, plays a role in cilia function (potential role in kidney function). Sequencing depth (17) ok, allele depth (12,5) imbalance?, GQ 99 ok, PL (139,0,336), PASS, **seems to be an accurate call, however the variant itself remains of unknown clinical significance + unknown Sift and polyphen scores.**
10. MAP3K15 (only found in Mother): GA heterozygous: this gene has not yet been associated with a certain disease or trait, it plays a role in apoptotic cell death triggered by cellular stresses. Sequencing depth (64) ok, allele depth (37,27) ok, GQ 99 ok, PL (831, 0,1224), PASS, **seems to be an accurate call, however the variant itself remains of unknown clinical significance + unknown Sift and polyphen scores.**

**FATHER**

Red (Polyphen and Sift scores above the threshold):

1. SERPINI1 (idem daughter): GA heterozygous: potentially pathogenic for familial encephalopathy with neuroserpin inclusion bodies, a progressive neurodegenerative disorder. Sequencing depth 43 ok, allele depth (23,20) ok, GQ 99 ok, PL (578, 0,732), VQSRTrancheSNP99.00to99.90, **Based on VQSR probably a false positive call**.
2. CCHCR1 (idem Daughter): GC heterozygous: this gene has not yet been associated with a certain disease or trait (except preliminary nevirapine hypersensitivity and psoriasis susceptibility), it encodes coiled-coil alpha helical rod protein 1. Sequencing depth: 32 ok, allele depth (15,17) ok, GQ 99 ok, PL (445,0,467), PASS, **seems to be an accurate call, however the variant itself remains of unknown clinical significance**.
3. ERCC6 (only found in Father): CG heterozygous: potentially related to a carrier status of Cerebrooculofacioskeletal syndrome, Cockayne syndrome, and/or UV-sensitive syndrome, disorders that affect bone and joint development and are characterized by extreme sensitivity to sunlight. Sequencing depth: 22 ok, allele depth (11:11) ok, GQ 99 ok, PL (364,0,359), PASS, **seems to be an accurate call, however the variant itself remains of unknown clinical significance**.
4. RHPN2 (idem Son and Daughter): TC heterozygous: this gene has not yet been shown to be associated with a genetic disease or trait, its protein product plays a role in the organisation of the actin cytoskeleton. sequencing depth (28) ok, allele depth (16, 12) ok, GQ 99 ok, PL (188, 0,220), VQSRTrancheSNP99.90to100.00, **based on VQSR, low GQ and allele imbalance probably a false positive call**.

Orange (Sift or Polyphen score above the threshold):

1. XDH (only found in Father): CA heterozygous: potentially related to a carrier status of Xanthinuria type 1, a metabolic disorder. Sequencing depth: 19 ok, allele depth (15,4) imbalance?, GQ 79 low, PL (79,0,536), PASS, **Based on low GQ and allele imbalance probably an inaccurate call**.
2. LAMA2 (only found in Father): AG heterozygous: potentially related to a carrier status of congenital merosin-deficient muscular dystrophy. Sequencing depth: 27 ok, allele depth (14;13) ok, GQ: 99, PL (438, 0,449), PASS, Sift 0,01; **seems to be an accurate call, however the variant itself remains of unknown clinical significance.**
3. SEC23B (only found in Father): GA heterozygous: potentially related to a carrier status of congenital dyserythropoietic anemia type 2, an inherited blood disorder. Sequencing depth 39 ok, allele depth (20,19) ok, GQ 99, PL (612,0,634), PASS; **seems to be an accurate call, however the variant itself remains of unknown clinical significance.**

Grey:

did not pass the Sift and/or Polyphen thresholds + no reliable homozygous calls

White (no Sift and/or Polyphen scores available): suspicious:

1. PYHIN1 (idem Son and Daughter): CT heterozygous: this gene has not yet clearly been correlated to a certain disease or trait, it functions as a tumor suppressor, in that respect down regulation of this gene is associated with breast cancer, sequencing depth: 22 ok, allele depth (15:7) imbalance, GQ 99 ok, PL (207, 0, 530), PASS, **based on imbalance in allele depth probably an inaccurate call, though GQ and VQSR seem ok**.
2. ALMS1 (idem Son): AG heterozygous: potentially related to a carrier status of Alström syndrome, a multi systemic disorder, characterised by childhood obesity, blindness, hearing loss and other symptoms. polyphen (0,985), sequencing depth (31) ok, allele depth (15,16) ok, GQ 99 ok, PL (539,0,459), PASS, **Seems to be an accurate call, however, the variant itself remains of unknown clinical significance + unknown Sift score**.
3. SMARCAL1 (idem Son & Daughter): CT heterozygous, potentially related to a carrier status of Schimke immuno-osseous dysplasia, characterised by short stature, kidney disease and a weakened immune system. Sequencing depth (25) ok, allele depth (12, 13) ok, GQ (99) ok, PL (397, 0,353), PASS, **seems to be an accurate call, however the variant itself remains of unknown clinical significance + unknown Sift and polyphen scores.**
4. MSH3 (only found in Father): AG heterozygous: this gene has not yet clearly been associated with a certain disease or trait, functions in the post-replicative DNA mismatch repair system. Defects in this gene may confer susceptibility to endometrial cancer. Sequencing depth: 19 ok, allele depth: 9;10 ok, GQ 99 ok, PL (342,0,291), PASS, Polyphen: 0,971, **seems to be an accurate call, however the variant itself remains of unknown clinical significance + unknown Sift score.**
5. CUL7 (idem Daughter): CG heterozygous, potentially related to a carrier status of 3-M syndrome type 1, a cause of dwarfism. Sequencing depth (12) rather low, allele depth (6,6) ok, GQ 99 ok, PL (193, 0,195), PASS, **seems to be an accurate call, however the variant itself remains of unknown clinical significance + unknown Sift and polyphen scores.**
6. RIMS1 (idem Daughter): GA heterozygous, potentially pathogenic for cone-rod dystrophy, autosomal dominant, a hereditary eye condition. Sequencing depth (28) ok, allele depth (12,16) ok, GQ 99 ok, PL (477,0,367), PASS, **seems to be an accurate call, however the variant itself remains of unknown clinical significance + unknown Sift and polyphen scores.**
7. SYNE1 (idem Son): GCTTG heterozygous: potentially related to a carrier status of autosomal recessive cerebellar ataxia type 1. Sequencing depth (49) ok, allele depth (20,29), GQ 99 ok, PL (1498, 0,797), PASS, **seems to be an accurate call, however the variant itself remains of unknown clinical significance + unknown Sift and polyphen scores.**
8. ABCA13 (only found in Father): CT heterozygous: This gene has not yet been associated to a certain disease or trait, it functions as a transmembrane transporter. Sequencing depth: 45 ok, allele depth (21,24) ok, GQ 99 ok, PL (791,0,638), PASS, Polyphen 0,874, **seems to be an accurate call, however the variant itself remains of unknown clinical significance + unknown Sift score.**
9. HOGA1 (idem Son & Daughter): CT heterozygous: potentially related to a carrier status of primary hyperoxaluria type 3, which can cause kidney damage. sequencing depth (15) ok, allele depth (6,9) imbalance?, GQ 99 ok, PL (277,0,189), PASS, **seems to be an accurate call, however the variant itself remains of unknown clinical significance + unknown Sift and polyphen scores.**
10. PNPLA2 (only found in Father): GT heterozygous: potentially related to a carrier status of Neutral lipid storage disease with myopathy, a metabolic disease characterised by muscle weakness, liver damage, heart problems and other complications. Sequencing depth: 15 ok, allele depth (8,7) ok, GQ 99 ok, PL (224,0,205), PASS, Polyphen 0,939, **seems to be an accurate call, however the variant itself remains of unknown clinical significance + unknown Sift score.**
11. SLCO1B3 (idem Son): TA heterozygous: this gene has been associated with Rotor type hyperbilirubinemia, it functions as a transporter in the liver and plays a role in bile acid and bilirubin transport. Sequencing depth (105) ok, allele depth (58,47) ok, GQ 99 ok, PL (1494,0,1836), PASS, Polyphen score 1, **seems to be an accurate call, however the variant itself remains of unknown clinical significance + unknown Sift score.**
12. EIF2B2 (idem Son & Daughter): AG heterozygous: potentially related to a carrier status of leukoencephalopathy with vanishing white matter, a progressive neurological disorder. Sequencing depth (86) ok, allele depth (42,44) ok, GQ 99 ok, PL (1484, 0, 1409), Polyphen score 0,948, PASS **seems to be an accurate call, however the variant itself remains of unknown clinical significance + unknown Sift score.**
13. PIF1 (idem Daughter): AC heterozygous: this gene has not yet been associated with a certain disease or trait, it functions as a DNA helicase that negatively regulates telomerase, a reverse transcriptase that maintains telomere length. Sequencing depth (9) rather low, allele depth (5,4) ok, GQ 83 rather low, PL (83,0,171), PASS, **seems to be an accurate call, however the variant itself remains of unknown clinical significance + unknown Sift and polyphen scores and rather low sequencing depth and GQ.**
14. HNF1B (idem Daughter): GT heterozygous: potentially pathogenic for MODY5 and/or renal cysts. Sequencing depth: 18 ok, allele depth (6;12) imbalance?, GQ 99 ok, PL (396,0,167), PASS, Sift 0,05, **seems to be an accurate call, however the variant itself remains of unknown clinical significance + unknown polyphen score.**
15. DNM2 (idem Son): CT heterozygous: potentially pathogenic for centronuclear myopathy, that affects skeletal muscles, or Charcot-Marie Tooth disease, a neurological disorder with progressive muscle weakness. sequencing depth (13) ok, allele depth (6,7) ok, GQ 99 ok, PL (196,0,147), PASS, **seems to be an accurate call, however the variant itself remains of unknown clinical significance + unknown Sift and polyphen scores.**
16. TMPRSS3 (only found in Father): AG heterozygous: potentially related to a carrier status of non syndromic deafness type 8 autosomal recessive, or Enteropeptidase deficiency, a metabolic disorder that causes severe diarrhea and poor growth. Sequencing depth (33) ok, allele depth (18,15) ok, GQ 99 ok, PL (489,0,612), PASS, **seems to be an accurate call, however the variant itself remains of unknown clinical significance + unknown Sift and polyphen scores.**
17. COL6A2 (idem Daughter & Son, daughter low GQ and depth): AAC heterozygous: potentially pathogenic for Bethlem myopathy or related to a carrier status of Ullrich congenital muscular dystrophy, sequencing depth: 15 ok, allele depth (6,9) ok, GQ 99 ok, PL (301,0,134), PASS, **seems to be an accurate call, however the variant itself remains of unknown clinical significance + unknown Sift and polyphen scores.**
18. XPNPEP3 (idem Son & Daughter): TTA heterozygous: potentially related to a carrier status of nephronophthisis-like nephropathy, a kidney disease. Sequencing depth (74) ok, allele depth (31,43), ok, GQ 99 ok, PL 1873,0,1150, PASS, **seems to be an accurate call, however the variant itself remains of unknown clinical significance + unknown Sift and polyphen scores.**
19. SHROOM4 (idem Daughter): TT homozygous (! gene is located on the X-chromosome, father is haploid and carries the Alt allele T): potentially related to a carrier status of X-linked mental retardation. Sequencing depth (34) ok, allele depth (0,34) ok, GQ 99 ok, PL (1323,102,0), PASS, **seems to be an accurate call, however the variant itself remains of unknown clinical significance + unknown Sift and polyphen scores.**

GeneTalk Report

Variant data of all exomes were also analyzed in GeneTalk with various filter settings [1]. For Father this analysis yielded variants in 13 genes, among them *CHI3L2*, *TPH1*, and *FPR1.* NM_004000: exon9: c.952C>T was reported to have a cis-regulatory effect on the expression of *CHI3L2*, a gene that might be involved in cartilage biogenesis but that has not been associated with any medical condition yet [2]. The heterozygous variant NM_004179: exon7: c.898G>A: p.A300T reduces the activity of tryptophan hydroxylase 1, *TPH1*, an enzyme that is required in the serotonin biosynthesis. Halmoy *et al.* analysed off-springs of mothers with an impaired serotonin production and showed that individuals score higher in average on an attention deficit/hyperactivity test [3]. In the Corpas family this is allele would be straightforward for Father to consult, as only female carriers would have the chance to affect their offspring. However, for Daughter that turned out to have this allele transmitted by Father, the genetic consult would be more challenging. In the community of geneticists there is growing support for communicating variants that might be health related and actionable. The ACMG Board published a list of *actionable* genes of which *TPH1* is not part [4], and it is questionable whether the medical advice to check serotonin levels especially during pregnancy would be reasonable given the current data. The variant NM_001193306:exon3:c.576T>G:p.N192K in *FPR1* might be of relevance whenever a carrier in the Corpas family requires some treatment with the immunosuppressant ciclosporin [5]. The variants in the remaining genes were classified as likely non-pathogenic polymorphisms or artifacts by the GeneTalk expert community, although they have been mentioned in the literature.

For Mother, filtering for heterozygous risk alleles yielded variants also in 13 genes. The only variant that’s of potential interest in a clinical context is the missense mutation R117W in *P2RX7* that affects protein function and might play a role in affective mood disorders [6]. However, in a genetic consultation about carrier risks, this allele would probably not be communicated as it is without any practical implications.

We then analysed the exome quartet assuming the son of the Corpas family was affected by some trait for which intended to find a genetic explanation. First, we filtered for possibly pathogenic homozygous variants that might cause recessive diseases:

- Functional filter: missense, nonsense, splicing, frame shifting
- Frequency filter: minor allele frequency <0.1 %
- Inheritance filter: recessive, homozygous in affected individual only

We did not find any potentially pathogenic variants with these settings and adapted the inheritance filter for recessive, compound heterozygous. This filtering approach is very powerful for identifying pathogenic alleles in individuals from non-consanguineous marriages that are affected by recessive disorders [7, 8] and yielded variants in altogether nine genes, *CELSR1, ZNF594, AHNAK2, ARHGEF17, MUC6, ZNF76, MUC4, ALPPL2,* and *NBPF10*. Most of these genes are known be highly variable or to cause sequencing artefacts due to pseudogenes and would thus not been considered as clinically relevant.

Finally we filtered for *de novo* mutations in Son, which is for instance a very promising filtering approach when looking for pathogenic candidate mutations in patients with non-syndromic intellectual disability [9]:

- Frequency Filter: remove all dbSNP entries
- Inheritance filter: set the status of the son to affected in the pedigree editor and filter for dominant.

We identified one *de novo* mutation, NM_022911:exon6:c.609C>G:p.F203L in *SLC26A6* that codes for an anion exchanger that might limit the rate of intestinal oxalate reabsorption [10]. The missense mutation is predicted to be pathogenic by MutationTaster and if Son will ever experience a renal colic from kidney stones, this is the genetics he might want to blame for it.

All analysis can also be reproduced in GeneTalk’s demo account with the quartet VCF of the Corpas Family.

1. Kamphans T, Krawitz PM: **GeneTalk: an expert exchange platform for assessing rare sequence variants in personal genomes.** *Bioinformatics* 2012, **28**:2515–2516.

2. Campino S, Forton J, Raj S, Mohr B, Auburn S, Fry A, Mangano VD, Vandiedonck C, Richardson A, Rockett K, Clark TG, Kwiatkowski DP: **Validating discovered Cis-acting regulatory genetic variants: application of an allele specific expression approach to HapMap populations.** *PLoS ONE* 2008, **3**:e4105.

3. Halmøy A, Johansson S, Winge I, McKinney JA, Knappskog PM, Haavik J: **Attention-deficit/hyperactivity disorder symptoms in offspring of mothers with impaired serotonin production.** *Arch Gen Psychiatry* 2010, **67**:1033–1043.

4. Green RC, Berg JS, Grody WW, Kalia SS, Korf BR, Martin CL, McGuire AL, Nussbaum RL, O'Daniel JM, Ormond KE, Rehm HL, Watson MS, Williams MS, Biesecker LG, American College of Medical Genetics and Genomics: **ACMG recommendations for reporting of incidental findings in clinical exome and genome sequencing.** *Genet Med* 2013:565–574.

5. Otani T, Ikeda S, Lwin H, Arai T, Muramatsu M, Sawabe M: **Polymorphisms of the formylpeptide receptor gene (FPR1) and susceptibility to stomach cancer in 1531 consecutive autopsy cases.** *Biochem Biophys Res Commun* 2011, **405**:356–361.

6. Roger S, Mei Z-Z, Baldwin JM, Dong L, Bradley H, Baldwin SA, Surprenant A, Jiang L-H: **Single nucleotide polymorphisms that were identified in affective mood disorders affect ATP-activated P2X7 receptor functions.** *J Psychiatr Res* 2010, **44**:347–355.

7. Kamphans T, Sabri P, Zhu N, Heinrich V, Mundlos S, Robinson PN, Parkhomchuk D, Krawitz PM: **Filtering for compound heterozygous sequence variants in non-consanguineous pedigrees.** *PLoS ONE* 2013, **8**:e70151.

8. Krawitz PM, Murakami Y, Hecht J, Krüger U, Holder SE, Mortier GR, Delle Chiaie B, De Baere E, Thompson MD, Roscioli T, Kielbasa S, Kinoshita T, Mundlos S, Robinson PN, Horn D: **Mutations in PIGO, a member of the GPI-anchor-synthesis pathway, cause hyperphosphatasia with mental retardation.** *Am J Hum Genet* 2012, **91**:146–151.

9. de Ligt J, Willemsen MH, van Bon BWM, Kleefstra T, Yntema HG, Kroes T, Vulto-van Silfhout AT, Koolen DA, de Vries P, Gilissen C, del Rosario M, Hoischen A, Scheffer H, de Vries BBA, Brunner HG, Veltman JA, Vissers LELM: **Diagnostic exome sequencing in persons with severe intellectual disability.** *N Engl J Med* 2012, **367**:1921–1929.

10. Jiang Z, Asplin JR, Evan AP, Rajendran VM, Velazquez H, Nottoli TP, Binder HJ, Aronson PS: **Calcium oxalate urolithiasis in mice lacking anion transporter Slc26a6.** *Nat Genet* 2006, **38**:474–478.
