# Supplementary material for: Crowdsourced direct-to-consumer genomic analysis of a family quartet
Source: BMC Genomics. 2015 Nov 7;16:910. doi: 10.1186/s12864-015-1973-7 (PMC4636840; doi:10.1186/s12864-015-1973-7)
Supplement: Additional file 4: — Consent form signed by the living participants of this study. (PDF 544 kb) [file 12864_2015_1973_MOESM4_ESM.pdf]

Participant Information Form Protocol
Based on Genomes Unzipped's consent form (http://www.genomesunzipped.org/)
Contact Person: Manuel Corpas
Website: http://manuelcorpas.com/
Description of Participants: Volunteers with expertise in areas related to the scientific, ethical, legal, social and commercial implications of genetic information, and their partners and family members.

What is the purpose of this form?
Before you elect to participate in the project, you need to understand if or how this project may affect you and your family. This form, along with other project documents available on the project, is intended to help you make an informed decision about your participation in this project. The project will be revised as needed, possibly on a frequent basis, and participants and prospective participants should check the website regularly to obtain the most current information about this project.

Why have you been asked to participate in this project?
You have been invited to participate because you (i) are an individual 21 years of age or older capable of making an informed decision to participate in the project, (ii) have expertise in an area related to the analysis of individual genetic data, or are the partner or family member, and (iii) have indicated that you would be willing to share information generated from your genome on a publicly accessible website.

I. PURPOSE

This project ("the Corpasome" project) intends to use the data provided by you and other family members both to perform scientific analyses and as a basis for communicating about genetics to a broader audience. Our specific goals are:

to shape informed discussion of genetic testing in both the lay and scientific communities, independent of commercial interests;
to foster a community of individuals interested in deeply exploring their own genetic data;
to generate tools for analysis of raw genetic data;
to provide independent, unbiased assessments of the technical validity and clinical utility of a variety of genetic testing products;
to explore ethical, legal, social and policy questions surrounding the acquisition, exploration and use of personal genetic data; and
to advocate change in areas where current legislation or research policies around genetic data are inadequate.

II. OVERVIEW
The Corpasome is a collaborative project involving experts in a variety of areas related to the implications and uses of personal genetic information. As a participant in this project, you will have the opportunity to obtain access to your own genetic information through a variety of genetic testing products. If you choose to participate by using one or more of these products, the information you receive will be made publicly available in a form that is linked to other personally identifying information, including your name, and will serve as the basis for analyses designed to test the uses and usefulness of individual genetic information. You will be encouraged to write about your experiences relating to this project on a custom-designed website, http://manuelcorpas.com. No efforts will be undertaken to anonymise your genetic or other data or to otherwise store, disseminate or analyse your data in a way that preserves or promotes privacy, anonymity or confidentiality. You should only participate if you are comfortable sharing genetic and genomic data, along with health and other trait data, with the public in a personally identified fashion.

The data provided by genetic testing companies is not intended to substitute in any way for professional medical advice, diagnosis or treatment, and it may not be used by you for any medical or clinical purpose unless the relevant sequence or other data, including any interpretations or findings you may receive as part of this project, are first confirmed at the direction of and in consultation with a licensed healthcare professional. You should be aware of the specific limitations and utility of each genetic test or other personalized service you participate in as part of this project.

We anticipate recruiting several core family members for the project.

Participation in this project is entirely voluntary. You do not have to participate in the project. You may withdraw your participation from this project at any time, according to the procedures and subject to the limitations more fully described in this consent form.

III. DURATION OF THE PROJECT AND YOUR PARTICIPATION

You will be deemed to be a participant from the time that you sign this consent form and submit it. Signing and submitting this consent form does not guarantee your full enrolment. Although signing the consent form means that you are a participant, your enrolment in the project is contingent upon the availability of resources and other relevant considerations as may be determined at its sole discretion and communicated to you.

There is no specified termination date for the project. While your participation is entirely voluntary and you may opt out at any time, analysis of your data may continue indefinitely following completion of your participation, unless you choose to withdraw your data or are removed from the project. Your ability to withdraw or remove data from the project is very likely to be limited, as described in more detail below.

IV. PRE-ENROLMENT PROCEDURES

4.1. If you have any living siblings who are your identical (monozygotic) twin, such sibling(s) will need to provide consent for your participation in this project before we will consider you for enrolment.

V. PARTICIPATION IN THE PROJECT

5.1. The core goal of this project is to explore the scientific, ethical, legal and social aspects of personal genetic testing through the analysis of publicly available genetic information from participants. As such, the project will aim to acquire genetic testing products, and these products will then be offered to participants who have agreed to make the resulting data publicly available through the project website, as well as to abide by the other conditions of participation in the project.

5.2. We will seek access to a variety of genetic testing products and services. Some of these may be commercially available at the time they are offered to participants; others may not yet be commercially available. While we will attempt to secure access to such products and services at no or low cost to participants, depending on availability and the project's resources, participants may be required to pay up to the full commercial cost of such products or services in order to participate in certain aspects of this project.

5.3. As new genetic testing products and services are obtained for the project, you may be given the option of participating in one or more analyses. If insufficient project resources are available for the provision of a new product or service to all members, the lead scientist, Manuel Corpas, in its sole discretion, will decide which members will be given the option of participating. You may be required to indicate whether or not you wish to participate prior to a specified deadline. If you indicate that you do not wish to participate in a specific analysis prior to the specified deadline you will not be prevented from participating in other future analyses.

5.4. Unless you specifically request otherwise, data from genetic testing products and services will be returned to you individually, and you will then have a chance to review the data before uploading it to the website.

5.5. The project will be seeking to obtain information on both common and rare genetic variants, some of which may have large effects on disease risk for you or your (actual or potential) children. The long- term goal of the project is to obtain complete genome sequences for each participant and to make those sequences publicly available online. You should agree to enter the project in the full knowledge that the goal of the project is to make your complete genetic information, along with other related health, trait and other data, freely available online.

5.6. At times you may be asked to supply phenotypic information, including information about your appearance, common variable traits such as lactose intolerance, and other non-genetic information such as family history of disease and other health data, environmental exposures or behavioural traits. Your participation in phenotypic data collection aspects of the project is entirely voluntary, and choosing not to volunteer this information will not affect your involvement in the project. Any such information that you choose to provide may be made publicly available.

5.7. By signing this consent form, you hereby agree that, by participating in this project you authorize Manuel Corpas to facilitate the public release of your genetic information, as well as certain other health, trait or other personal data voluntarily submitted by you, through the http://manuelcorpas.com publicly accessible website and database, or

in such other formats and/or locations as may be designated, without your further consent. You hereby acknowledge the risks associated with the receipt, return and/or public release of such data and information.

VI. ONGOING PARTICIPATION FOLLOWING ENROLLMENT

6.1. Additional personal and trait information may be requested and submitted by you on an entirely voluntary basis. Any additional information disclosed by you may be made publicly available via the public website and database without your additional consent.

6.2. Tissue samples, such as buccal swabs, skin swabs, hair samples or saliva samples may be collected by you or by third parties, including providers of genetic testing or other services. We will not collect or directly analyse tissue specimens, but information obtained from such samples and provided by you to the project may be incorporated into the project, with the results made publicly available via the public website and database.

6.3. Family members will generate analytical results deriving from the information you have provided to the project, and will make these results available along with your other information already on the public website and database. Scientists performing analyses are not obliged to contact you directly or seek additional consent for any analysis performed using your data, or for the release of the data online.

6.4 The Corpasome project may generate publications based on analyses of participant data. Participation in the project is not a guarantee that you will be given authorship on publications arising from the project; authorship status will be based on your degree of participation in the relevant analyses or the writing of the manuscript. We will not seek additional consent from you prior to publishing results from analysis of your data.

6.5. As a member of this project you will have access to certain genetic, phenotypic and other data from other participants. In some cases your degree access may exceed the access provided to the public at large. Although (as stated above) you are not obliged to contact or seek additional consent from other participants. Before analysing their data or publishing your results, you are strongly encouraged to consider the impact of your findings on other participants and to discuss the implications of any unexpected or potentially damaging results with such individuals prior to making the results of any such analyses available to the public.

6.6. We may periodically circulate a "Participant Questionnaire" to all participants and request that each participant answer certain questions about their experience with the project. Examples of such questions include:

a. What negative and/or positive events have happened to you, your spouse and/or your relatives or acquaintances due to your participation in this project?
b. What are the reactions or responses of your relatives and acquaintances to the posting of your genetic, trait and other data?
c. Please report incidents of being contacted by acquaintances or by strangers (including researchers, health care providers or members of the media) regarding your data being posted online.
d. In what ways has this project positively or negatively influenced your interactions with your medical care providers or your receipt of or access to health care services?
e. Has your involvement in this project triggered any medical work up, investigations, or treatments that would not otherwise have been done? If you answer yes, please describe (i) the specific medical intervention and (ii) the findings or consequences of the medical intervention with regard to your health. Medical work up that would have been performed had you not participated in this project, whether due to symptoms, a personal or family medical history, routine screening or any other reason, should not be included.

You will be asked to provide written answers to any Participant Questionnaire – or a "no change" reply – within two weeks of your receipt of such questionnaire. Whether or not you have received a questionnaire, you should immediately report any material differences between their experiences as an enrollee and the contents of this consent form or other project documents.

You are also encouraged to discuss your experiences as a participant with other participants. If at any time you are concerned that your participation in the project may have an adverse effect on you, your spouse, your relatives and/or your acquaintances you should immediately notify Manuel Corpas.

VII. RISKS AND DISCOMFORTS

This project represents a new and experimental form of discussion about genetics, as a result, it is impossible to accurately predict all of the possible risks and discomforts that you might experience as a result of your participation in this project. In this section you will read about the risks that we have identified as potentially relevant to your participation in this project.

You are strongly encouraged to think carefully about these risks, as well as any other risks or discomforts that you anticipate might arise as a result of your own unique circumstances. These might include your own health or medical conditions, your family and personal relationships or any other factor that is specific to you. In addition to understanding the risks outlined in this Section VII, you should feel confident that you have sufficient knowledge of genetics, human subjects research and the benefits and risks of participation in this project to make an informed decision about whether participation is right for you.

You are strongly encouraged to discuss this project and its potential risks, as outlined below and on the project website, with your immediate family members as well as with your physician and/or other qualified health care providers. You are also encouraged to discuss with the Principal Investigator directly any additional concerns that you may have regarding the risks to you of participating in this project.

Finally, because the science in this area is evolving, and data will be collected on an ongoing basis, the risks involved due to your participation in this project, as well as the likelihood and severity of such risks, will change over time. You will not be asked to review and re-sign this consent form every time new information related to the risk of participation becomes available. You are advised that the website will be revised, possibly on a frequent basis, and participants and prospective participants should check the website regularly to obtain the most current information regarding potential risks and discomforts as they become apparent.

Please remember that you are free not to participate if you have not already enrolled, and to withdraw at any time if you have already enrolled in the project.

7.1. The risks of public disclosure of your genetic, trait and other personal information could affect your employment, insurance and financial well-being and social interactions for you and your immediate family. The following is a non-comprehensive list of hypothetical scenarios that could pose risks for you and/or your family:

a. Data that you provide (such as facial images, other trait data or genetic data) may be used to identify you, resulting in higher than normal levels of contacts from the press and other members of the public motivated by positive or negative feelings about the project. This could mean a significant loss of privacy and personal time.

b. Anyone with sufficient knowledge and resources could take your genetic data and/or posted trait information and use that data, with or without modification, to (i) infer paternity or other features of your genealogy, (ii) claim statistical evidence that could affect your or one of your family member's employment, insurance or ability to obtain financial services, (iii) claim relatedness to criminals or incriminate relatives, (iv) make synthetic DNA and plant it at a crime scene, or otherwise use it to falsely identify you, or (v) reveal the possibility of a disease or unknown propensity for a disease.

c. Whether or not it is lawful to do so, you and/or a member of your family could be subject to actual or attempted employment, insurance, financial or other forms of discrimination or negative treatment on the basis of the public disclosure of your genetic and trait information by a third party. Although some countries have laws that prohibit certain forms of genetic discrimination, these laws may not apply to you, may not protect against all forms of discrimination or may not stop a third party from discriminating against you even where it is prohibited by law.

d. If you have previously made available or intend to make available genetic information in a confidential setting, for example in another research project or in a clinical trial, the data that you provide as part of your participation may be used, on its own or in combination with your previously shared data, to identify you as a participant in otherwise confidential genetic research or trials. This means that any data or other information you may have shared pursuant to a promise of confidentiality or privacy may become public despite your intent that it be kept private and confidential. Depending on the nature and context of the data or information, this could result in certain adverse effects for you, including ones not contemplated by this consent form.

7.2. Your genetic data, trait data and other information related to you that is made publicly available, while directly associated only with you, may also have relevance to your family members. Although in many instances any conclusions that may be inferred from your publicly available information may be speculative with respect to you, and even less predictive with respect to your family members, the complete set and magnitude of the risks that the public availability of this information poses to you and your relatives is not known at this time. You are strongly encouraged to discuss this project and its potential risks, including the fact that not all risks are known, with your immediate family members.

7.3. You may have family members or partners who have already contributed genetic data or who plan to do so in the future. In such cases you should consider the additional risks and implications. For instance, if you and your partner are both participants you may learn that both of you are carriers for recessive disease-causing variants in the same gene, suggesting a higher risk of severe disease in your children.

7.4. Unintended Disclosure. If you are enrolled, receive and review genetic or other data and decide not to make that data available through the website, you should be aware that the public disclosure of such data due to unintended data breaches, including hacking or other activities outside of the procedures authorized, is still possible. Should this occur, you would be subject to the various risks and discomforts described in this Article VII and throughout this consent form.

7.5. If you choose to make your genetic data and other information available it will be published via the project's publicly accessible website and database, and it will be available to third parties without legal restriction. As a result, neither you nor the project will be able to control or restrict the access, use, reproduction, modification or analysis of your data and other public information. Your data and other public information may be made public in other forms beyond its inclusion in the database. It may also be altered or modified, without either your or the project's consent, in a way that might be inaccurate and/or upsetting to you. For instance, a third party could access your publicly available sequence data, alter it and republish it to suggest that you had a propensity for a disease or other detrimental trait. Additional adverse effects are also possible.

7.6. You are advised that the project is unable to guarantee the accuracy or the validity of any data provided to you by the providers of genetic testing products or services, by other participants or by other third parties. The data provided to you by genetic testing providers, along with any interpretation of that data provided by such providers, or by other participants or any other third party, is not a suitable substitute for professional medical or clinical advice, diagnosis or treatment, and may not be used by you for any medical or clinical purpose unless the relevant sequence or other data are first confirmed at the direction of and in consultation with a licensed healthcare professional.

a. Comprehensive screening of genetic data for pathogenic genetic variants is not done clinically or routinely at this time. The clinical importance of even well established pathogenic variants that are found through this type of screening is not known with certainty at this time. Furthermore, although there is considerable information discussing possible connections between genetic information, such as may be disclosed by your genetic data, and clinical or medical outcomes, some of these connections, especially when screened for in the general population, remain uncertain, subject to further research and neither their validity nor their clinical usefulness can be confirmed by at this time.

b. Regardless of any specific interpretations or findings provided in or omitted from your personal genetic data, you are likely to be subjected to additional interpretations - both accurate and inaccurate - of your public data by outside sources.

c. Knowledge of potentially detrimental genetic variants may cause anxiety. As a result, you may be motivated to seek Health or Medical Care, as defined below, to verify the accuracy of such interpretations, whether provided by the project or by other sources. Should you choose to pursue such Health or Medical Care you could be exposed to additional risks and/or discomforts, several of which are identified below.

7.7. The project will not (i) provide you with, (ii) arrange for, (iii) pay for, reimburse you for or otherwise subsidize or (iv) provide you, your physician or any other health care provider with any recommendations, advice, counselling or other guidance with respect to, any of your Health or Medical Care. For purposes of this consent form, “Health or Medical Care” means both (i) current medical or clinical advice, counselling, diagnosis or treatment, preventative action or other related course of action of any kind and (ii) follow up clinical or medical advice, counselling, diagnosis or treatment, preventative action or other related course of action of any kind.

a. The project is not responsible for any aspect of your Health or Medical Care, including, without limitation, accurately predicting disease or disease risk, informing you of pathogenic sequence

variants, or providing you with accurate or valid genetic data or interpretations of your genetic data, or any other data. No Health or Medical Care will be made available and, as described above, no special arrangements, for compensation or otherwise, will be made should you require or choose to pursue any Health or Medical Care as a result of your participation in the project.

b. You should seek the advice of your physician or other qualified health care provider if you have questions regarding any information provided to you, including with respect to your genetic data or interpretation by the project's participants or other third parties. You should not ignore professional medical advice from your doctor or any other qualified health care provider on the basis of any information contained or not contained in reports provided to you by the project or any third party, and you should not interpret your genetic data or interpretations provided by the project participants or other third parties as recommending or discouraging any specific treatment plan, product or course of action with respect to your Health or Medical Care.

c. In the event that you, in conjunction with your physician or other qualified health care provider, decide that any change in your Health or Medical Care is necessary or advisable as a result of any information you obtained as a participant, you (or your third party payer, if applicable) will be solely responsible for all resulting payments and costs associated with such Health or Medical Care.

d. Any Health or Medical Care that you may determine, after consultation with your physician or other qualified health care provider, is necessary, whether as a result of your participation in this project or otherwise, may be invasive and have its own associated risks and expenses. Serious risks, including death, may be involved in any such Health or Medical Care. You should carefully consider these risks, as well as whether or not you have access to the financial and other resources to pursue such Health or Medical Care.

e. In the event that your physician or other qualified health care provider is directly or indirectly involved with the project, any Health or Medical Care that you receive from such provider, including medical or other advice, counselling or clinical management, represents Health or Medical Care provided by such provider pursuant to your existing physician-patient relationship, and is not to be construed or interpreted as the provision of Health or Medical Care.

7.8. If physical injury resulting from participation in this project should occur, please seek medical care immediately and contact the Principal Investigator. Although, as described above, the project will not normally provide any Health or Medical Care to participants it may, in rare instances and at its sole discretion, seek to arrange Health or Medical Care in the event of an emergency. Should this occur, you (or your third party payer, if applicable) may be billed for the cost of such Health or Medical Care.
Should the project make any Health or Medical Care available neither it, nor any individual associated with it, are admitting any fault or liability for any injury that you may have suffered.

VIII. BENEFITS
8.1. There are no proven benefits to you from your participation in this project.

IX. INTELLECTUAL PROPERTY
9.1. One of the goals of the Corpasome project is to encourage public access to genetic information, and promote collaboration in the analyses of that information. In order to aid in the

development of analytical tools and interfaces for scientists, doctors, and individuals around the world, the project will endeavour to develop data structures and other tools, including legal agreements, to maximize the ability of the project to share its data in the broadest possible fashion.

9.2. The Corpasome will not claim ownership of any information or data that you provide directly to the project as part of your participation. However, as a condition of your participation you grant the project and any person the right to use such information or data without restriction including, without limitation, copying, distributing, transmitting, publicly displaying, publicly performing, publishing, reproducing, editing, translating, reformatting, and creating derivative works, subject only to your ability to withdraw from the project, as that ability is described in this consent form. Any information, data or materials created or prepared by Manuel Corpas from the information or data you contribute, as well as the results of any analysis performed by or in collaboration with other scientists, are in the public domain and not owned by you. However, consistent with the goals of the project and this consent form, we will attempt to make all information, data or other materials relating to the project, and to which it has appropriate legal rights, available to both you and the public as described in this consent form, subject to such conditions or limitations as it may determined consistent with this consent form. Manuel Corpas is unable to guarantee if, when or in what form you will receive access to any information, data or materials as part of your participation in this project.

9.3. Other than for purposes of cost recovery, neither Manuel Corpas nor manuelcorpas.com will license or otherwise make available your DNA samples, genetic data, and personal information to any person, institution, company or other third party for the financial gain or commercial profit. However, information and materials that you provide, including genetic data and other information, will be made available to third parties for research, clinical or therapeutic, commercial or other purposes, and these third parties may commercially profit from the data or other information that you contribute to the project.

9.4. You will not be compensated for your participation in the project. Neither you nor your heirs will gain financially from any discoveries, whether or not of a commercial nature, made using the information and/or specimens that you provide.

X. CONFIDENTIALITY

10.1. If you are enrolled in this project, your genetic and trait information will not be maintained or made available in a confidential, private or anonymous fashion. Your genetic and trait information will be made available via a publicly accessible website and database, according to the procedures described above. Public disclosure of your information due to unintended data breaches, including hacking or other activities outside of the procedures described above, is also possible.

10.2. Your genetic and trait data will not be intentionally sent directly to your health care provider. However, because this information will be publicly available, and will be identified as yours, it could become part of your medical record or be shared with your health care provider or provided to others due to the activities of one or more third parties.

10.3. Your reply to any Participant Questionnaires will be confidential by default, unless you specify otherwise. However, governmental agencies or project sponsors may request or require this information in order to judge the risks to you and any other project participants and we will share your replies to any Participant Questionnaires with such entities to the extent required or reasonably requested.

Responses to the Participant Questionnaires that may impact other participants or the public generally may be more widely shared. Such responses will be paraphrased and/or will have all information reasonably likely to identify you removed prior to making this information publicly available on the public website or elsewhere for purposes of public education or risk management. If you would like your answers to be identified as yours, then you will need to indicate that as part of your response to the Participant Questionnaires. Although we will take reasonable steps to ensure that your responses to Participant Questionnaires, if published, are not identified as yours without your consent, we are unable to guarantee the anonymity of your responses.

10.4. The results of this project may be published in a medical book, journal, website or webpage, or used for teaching purposes. Your name and other identifiers (such as your photograph and medical information provided during the course of your participation in the project) may be used in such publications or teaching materials. You will not be notified prior to such use.

XI. REFUSAL OR WITHDRAWAL OF PARTICIPATION

11.1. Participation in this project is voluntary. You do not have to participate in this project. You may withdraw your participation and/or your data from this project at any time, as described in this consent form, and you need not provide a reason.

11.2. You are free to decide at any time that you no longer want your genetic, trait or other data to be used as part of this project, but you are advised that there are significant limitations on your ability to prevent the future use of such data and/or information.

11.3. If you choose to withdraw from the project and request that your genetic, trait or other data be removed, within 6 weeks we will delete all such specified data pertaining solely to you, and issue requests to any organizations or researchers with whom the Corpasome has any formal data sharing agreements to likewise delete such data within a reasonable time frame. You are advised, however, that once any data or information obtained about you during the course of your participation in the project is posted on the Internet or otherwise made available, other organizations and individuals who have no formal data sharing agreement with the Corpasome may acquire copies of it, and there will be no mechanism to ensure that they delete their copies or for us to even know what copies may exist. If you choose to withdraw from the project, we will not remove your information from ongoing or completed research or studies that utilize such information.

11.4. We may decide, at its sole discretion, to end your participation in this project at any time. If this project terminates your participation it will provide an explanation for its doing so. If your participation is terminated by us, you may request that the project delete any or all of your data in accordance with the provisions set forth above.

11.7. In order to comply with the terms of this consent form, as well as with legal or other requirements, we may continue to maintain certain information about you following the conclusion of your participation in this project, including your name, date of first participation, date of enrolment and date of termination or withdrawal.

XII. ALTERNATIVES TO PARTICIPATION

12.1. The alternative is not to participate in the Corpasome project.

XIII. PROJECT-RELATED CONTACT INFORMATION:
13.1. If you have any questions or concerns about the project you should contact:

Manuel Corpas
Email: mc@manuelcorpas.com

SIGNATURE
I have read this entire participant information form and I understand it completely. I confirm that I understand the purpose of this project, the procedures to be carried out, the possible risks and discomforts of participating in this project, the potential benefits that I may experience, and the alternatives to my participation in this project. All of my questions have been answered to my complete satisfaction. I understand that by signing this form I am acknowledging and agreeing to all of the terms and conditions of participation set forth above.

Printed Name:

Date:
Signature:
